# Medicinal food plants of Sabah (Eastern Malaysia): a source of potential natural products and nutraceuticals for the fight against cancer

**DOI:** 10.1080/13880209.2026.2663269

**Published:** 2026-05-07

**Authors:** Carynn Tanbuda, Mazdida Sulaiman, Pauline Yong Pau Lin, Fiffy Hasnidah Saikim, Nor Azizun Ruzdi, Mogana Rajagopal, Nicholas Pang Tze Ping, Nor Hayati Abdullah, Jhonnel Villegas, Sanen Marshall, Mukesh Singh Sikarwar, Veeranoot Nissapatorn, Prapairat Seephonkai, Marcelo Iriti, Mark S. Butler, Christophe Wiart

**Affiliations:** ^a^ Institute for Tropical Biology and Conservation, Universiti Malaysia Sabah, jalan UMS, Kota Kinabalu, Sabah, Malaysia; ^b^ Department of Chemistry, Faculty of Science, Universiti Malaya, Kuala Lumpur, Malaysia; ^c^ Faculty of Social Sciences and Humanities, Universiti Malaysia Sabah, Jalan UMS, Kota Kinabalu, Sabah, Malaysia; ^d^ Faculty of Pharmaceutical Sciences, UCSI University, Cheras, Kuala Lumpur, Malaysia; ^e^ Faculty of Medicine, Universiti Malaysia Sabah, jalan UMS, Kota Kinabalu, Sabah, Malaysia; ^f^ Natural Product Division, Forest Research Institute of Malaysia, Kepong, Kuala Lumpur, Malaysia; ^g^ Faculty of Teacher Education, Davao Oriental State University, Davao Oriental, Philippines; ^h^ Faculty of Tropical AgriSciences, Czech University of Life Sciences Prague, Praha-Suchdol, Czech Republic; ^i^ Center for the Promotion of Knowledge and Language Learning, Universiti Malaysia Sabah, jalan UMS, Kota Kinabalu, Sabah, Malaysia; ^j^ Amity Institute of Pharmacy, Amity University, Gwalior, Madhya Pradesh, India; ^k^ Futuristic Science Research Center (FSRC) - School of Science, Research Excellence Center for Innovation and Health Products (RECIHP) and World Union for Herbal Drug Discovery (WUHeDD), Walailak University, Nakhon Si Thammarat, Thailand; ^l^ Department of Chemistry, Faculty of Science, Mahasarakham University, Maha Sarakham, Thailand; ^m^ Department of Biomedical, Surgical and Dental Sciences, University of Milan, Milan, Italy; ^n^ MSBChem Consulting, Brisbane, QLD, Australia

**Keywords:** Borneo, ethnopharmacology, natural products, oncology

## Abstract

**Context:**

The pharmaceutical and nutraceutical industries are seeking structurally and pharmacologically novel anticancer molecules, as well as onco-protective nutraceuticals. One approach to achieving this goal is to study traditional pharmacopoeias, particularly those from regions where cancers are less common. Certain ethnic groups in Sabah (East Malaysia) appear to have a low incidence of cancer, and the study of their pharmacopeia could lead to the discovery of original anticancer molecules or nutraceuticals.

**Objectives:**

This review presents a selection of 64 plants used for medicinal food in Sabah their potential for clinical uses.

**Methods:**

The data for this focused narrative review were gathered from Google Scholar, PubMed, ScienceDirect, Web of Science, PubMed, the Internet Archive, and Google books. For each plant the search string included the binomial denomination and the words “cytotoxic” or “tumors.” of the binomial denomination of each plant and “cytotoxic” and “tumors” was employed. Each result was examined and articles that did not contain information relevant to the topic or coming from non-peer-reviewed journals were excluded.

**Results:**

Eight plant species, of which *Helminthostachys zeylanica* (L.) Hook., *Pycnarrhena tumefacta* Miers, *Myrmecodia platytyrea* Becc., and *Mangifera pajang* Kosterm, demonstrate activities *in vitro* and *in vivo*, which call for further research. Others constitute a source of cytotoxic natural products that warrant further investigation.

**Conclusion:**

There is currently a need to find oncopreventive nutraceuticals as well as original natural products for developing anticancer drugs. Such products could potentially be found among the medicinal and edible plants of Sabah. Further studies are needed.

## Introduction

Cancer is the second leading cause of death in most regions of the world (Bray et al. 2021), and was responsible for approximately 10 million deaths in 2020 (WHO 2022). By 2050, the number of cancers worldwide will reach 35.3 million, with developing countries being the most affected (Bizuayehu et al. 2024). Although some cancers are treatable when diagnosed early, the prognosis for some remains poor, and it is more necessary than ever to find anticancer drugs.

Several anticancer drugs originate from the plant kingdom. These tumoricidal compounds include vinblastine (Hodgkin’s lymphoma), podophyllotoxin (breast cancer), paclitaxel (Taxol) (ovarian and breast cancer), and homoharringtonine (leukemia) (Yang et al. 2025). These substances, derived from the secondary metabolism of plants, have been used in the semi synthesis of anticancer drugs such as vinorelbine (breast and ovarian cancer) from vinblastine, cabazitaxel (prostate cancer) from paclitaxel (Yang et al. 2025), and Sacituzumab Tirumotecan (breast cancer) from camptothecin (Butler et al. 2026). Many semi-synthetic camptothecin derivatives are the subject of clinical trials (Butler et al. 2026) including ABBV-969, for the treatment of prostate cancer (Tolcher et al. 2025).

Another important aspect in the fight against cancer is prevention, or what could be called “onco-prevention”. It is known that some cancers develop after chronic exposure to carcinogenic substances such as 7,12-dimethylbenz[*a*]anthracene (tobacco, industrial chemicals) (Rodgman and Perfetti 2006), glyphosate (food) (Wogan et al. 2004; Bai and Ogbourne 2016), as well as through chronic inflammation (Multhoff et al. 2011), oxidative stress (Jelic et al. 2021), and in cases of immunodeficiency (Pai et al. 2021). Therefore, consuming medicinal foods or plants with antimutagenic, anti-inflammatory, and/or immunostimulatory properties could be a way to prevent cancer or even extend the life expectancy of patients.

In some communities, the incidence of cancer is low, or even non-existent, even among the elderly. This has been observed in Sudan, Gambia, Sri Lanka, and among American Indians (Levin 1910). This phenomenon also appears to exist in Sabah (Eastern Malaysia) among local populations belonging to Bornean-speaking ethnic groups, including the Dusun, Kadazan and Murut, as well as among non-Bornean-speaking ethnic groups such as the Lundayeh, Brunei and Rungus (King and King 1984, Kroeger and Kroeger 1986; Wiart 2024; Tanbuda et al. 2025). These ethnic groups are distributed across areas of high biodiversity and high rates of plant endemism (Beaman and Beaman 1990). To date, approximately 696 species of medicinal plants have been recorded in Sabah, including a significant number of endemic species.

In this context, this review aims to present 64 species of medicinal food plants from Sabah whose phytochemical study could lead to the development of anticancer drugs or whose consumption in the form of nutraceutical products could prevent the appearance of cancer or allow people to live longer with cancer.

## Methodology

In a previous study, we reviewed the 696 species of medicinal plants in Sabah, analyzing their taxonomical distribution and utilization among ethnic groups (Tanbuda et al. 2025). Out of these species we selected 64 medicinal food plant species for which there are few or no phytopharmacological studies and which belong to families known to produce cytotoxic natural products. This study is a focused narrative review, based on structured literature searches conducted for each selected species. The approach aims to provide an integrated overview of ethnobotanical uses, phytochemistry, and pharmacological activities, rather than meeting the criteria of a formal systematic review. For each plant covered, a search was conducted using Google Scholar, PubMed, ScienceDirect, Web of Science, Internet Archive, and Google Books. The search string included the binomial denomination of each plant and the words “cytotoxic” or “tumors.” If entering a binomial denomination and the words “cytotoxic” or “tumors” did not yield any results, the process was repeated with the genus name. The inclusion criteria focused on articles, conference proceedings, and books specifically addressing the selected medicinal food plant species. Exclusion criteria included articles, conference proceedings, and books related to medicinal plants from regions or countries outside Sabah, as well as non-English works or those not peer-reviewed.

## Plants used by multiple ethnic groups

### General observations

Most of the plants employed for medicinal purposes by multiple ethnic groups are not endemic, except for some of those used by both Dusun and Kadazan (Table 1 and 2).

**Table 1.** Medicinal plants of Sabah.

[Subclass]	Genus, species, authority	Symptoms/diseases (ethnic group)	local names	(references)
**[Superorder]**				
**Order**				
Family				
**[Lycopodiidae Bek. (1862) (Lycophytes)]**				
Selaginellales Prantl (1854)				
Selaginellaceae Willk. (1854)	*Selaginella argentea* (Wall. ex Hook. & Grev.) Spring^†ψ^	Asthma, body aches, fever, headaches (Murut)	sondotnulogo	(Ahmad and Raji 19 1992; Kulip 2003)
		Medicinal (Bonggi-Molbog)	ipa ipa puteh	(Lin 2022)
**Ophioglossidae Klinge (1832)**				
**Ophioglossales Link. (1833)**				
Ophioglossaceae Martinov (1820)	*Helminthostachys zeylanica* (L.) Hook.	Cancer, wounds (Lundayeh)	pajerok	(K Kulip 2007)
		Postpartum (Bonggi-Mogbol)	onitug	(Lin 2022)
		Medicinal food (Dusun)	aruk aruk	
**[Polypodiidae Cronquist, Takht. & W. Zimm. (1966)]**				
**Blechnales Pic. Sem. ex Reveal (1993)**				
Blechnaceae Newmann (1844)	Stenochlaena palustris (Burm. f.) Bedd.	Medicinal food, postpartum, fever, skin diseases (Dusun)	lambiding	(Maid et al. 2017)
		Postpartum, fever, skin diseases (Kadazan)	lambiding	
Gleicheniales Link (1825)				
Gleicheniaceae C. Presl (1825)	*Gleichenia truncata* (Willd.) Sprain^†ψ^	Sore eyes (Dusun)	laputong	(Kulip 2014)
**Polypodiales Link (1833)**				
Athyriaceae Alston (1956)	*Diplazium cordifolium* Bl.^†ψ^	Cold, fever (Bajau)	giman	(Wiart 2024)
	*Diplazium esculentum* (Retz.) Sw.^ψ^	Medicinal food (Dusun)	pakis	(Maid et al. 2017)
Polypodiaceae Link J. Presl & C. Presl (1822)	*Drymoglossum piloselloides* (L.) C. Presl^†ψ^	Diuretic, gallstones, hypertension (Brunei)	sisik naga	(Mahmud and Razali 2016)
	*Drynaria sparsisora* (Desv.) T. Moore ^ψ^	Asthma, heart diseases (Dusun)	tapako	(Wiart 2024)
		Asthma, heart diseases (Kadazan)	tapako	(Wiart 2024)
Schizaeales Schimp. (1869)				
Lygodiaceae M. Roem. (1840)	*Lygodium circinnatum* Sw.^†ψ^	Venereal diseases (Lundayeh)	waratang	
		Womb diseases (Dusun)	taribu mianai	(Voeks and Nyawa 2 2006)
Nephrolepidaceae Pic. Serm. (1975)	*Lygodium salicifolium* C. Presl^†ψ^	Chickenpox, smallpox (Lundayeh)	ubat amur	
Pteridaceae E.D.M. Kirchn. (1831)	*Nephrolepis acutifolia* (Desv.) Christ ^ψ^	Medicinal food (Dusun)	paku puteh	
	*Acrostichum aureum* L.	Medicinal food (Dusun)	paku besar	
**[Gnetidae Pax (1894)]**				
**Gnetales Blume (1835)**				
Gnetaceae Blume (1833)	*Gnetum macrostachyum* Hook.f.^ψ^	Fatigue, postpartum (Brunei)	kokos	(Kulip 1997)
**[Magnoliidae Novák ex Takht. (1967)]**				
**(Austrobaileyanae Doweld ex M.W. Chase & Reveal (2009))**				
**Austrobaileyales Takht. ex Reveal (1992)**				
Schisandraceae Blume (1830)	*Kadsura borneensis* A.C. Sm^†ψ^	Cramps (Lundayeh)	putu urat	
	*Kadsura lanceolata* King^†ψ^	Bacterial skin infection, swelling (Dusun)	topis	(Wiart 2024)
**[Magnolianae Takht. (1967)]**				
**Laurales Juss. ex Bercht. & Presl (1820)**				
Lauraceae Juss. (1789)	*Eusideroxylon zwageri* Teijsm. & Binn.^†ψ^	Blow-gun darts poison (Murut)	belian	(Kulip 2003)
	*Litsea garciae* Vidal. ^ψ^	Dislocation, sprains (Murut)	pengolaban	(Wiart 2024)
		Medicinal food (Dusun)	pengolaban	(Maid et al. 2017)
**Magnoliales Bromhead (1838)**				
Annonaceae Juss. (1789)	*Artabotrys roseus* Boerl.^†ψ^	Medicinal (Dusun)	gangon	
		Medicinal (Kadazan)	gangon	
	*Goniothalamus roseus* Stapf^†ψ^	Fatigue, fever (Dusun)	limpanas	(Foo et al. 2016)
	*Goniothalamus velutinus* Airy Shaw ^ψ^	Magic rituals (Dusun)	kalampanas	(Voeks 2007)
	*Goniothalamus woodii* Merr ^†ψ^	Magic rituals (Murut)	tampaliu	(Wiart 2024)
	*Polyalthia tenuipes* Merr.^†ψ^	Sick children (Dusun)	kabanking	(Wiart 2024)
**[Lilianae Takht. (1967)]**				
**Commelinales Mirb. ex Bercht. & J. Presl (1820)**				
Eriocaulaceae Martinov (1820)	*Eriocaulon longifolium* Nees ex Kunth^†ψ^	Canker sores (Dusun)	kumpau sambangau	(Voeks and Nyawa 2006)
Poaceae Barnhart (1895)	*Dendrocalamus asper* (Schult. f.) Backer ex K. Heyne	Medicinal food (Dusun)	buluh betong	
	*Garnotia acutigluma* (Steud.) Ohwi^†ψ^	Venereal diseases (Lundayeh)	udu bulu	
	*Panicum palmifolium* J. Koenig^†ψ^	Malaria (Dusun)	tandaki	(Maid et al. 2017; W Wiart 2024)
		Malaria (Kadazan)	tandaki	(Wiart 2024)
Zingiberaceae Martinov (1820)	*Boesenbergia pulchella* (Ridl.) Merr.^†ψ^	Skin diseases (Dusun)	lipat	(Kulip 2007)
		Skin diseases (Kadazan)	lipat	(Kulip 2007)
	*Plagiostachys albiflora* Ridl.^†ψ^	Medicinal food (Dusun)	wongking	(Kulip 2007)
	Etlingera elatior (Jack) R.M. Sm.	Fever, flatulence, medicinal food (Dusun)	topu	(Kulip 2007a)
		Fever, flatulence (Kadazan)	topu	(Kulip 2007a)
**[Ranunculanae Takht. ex Reveal (1992)]**				
**Ranunculales Juss. ex Bercht. & J. Presl (1820)**				
Menispermaceae Juss. (1789)	*Pycnarrhena tumefacta* Miers^†ψ^	Bacterial skin infection (Lundayeh)	fatagah	
		Medicinal food (Murut)	apa	(Wiart 2024)
		Medicinal food (Dusun)	apak	
		Medicinal food (Kadazan)	apak	
**[Rosanae Takht. (1967)]**				
**Vitales Juss. ex Bercht. & J. Presl (1820)**				
Vitaceae Juss. (1789)	*Ampelocissus polita* (Miq.) Pelser^†ψ^	Medicinal food (Dusun)	mban ambuk	(Voeks and Nyawa 2006)
**Fabales Bromhead (1838)**				
Fabaceae Lindley (1836)	*Airyantha borneensis* (Oliv.) Brummitt^†ψ^	Fatigue (Dusun)	barayung	(Voeks and Nyawa 2006)
		Fever, hypertension, toothaches (Murut)	matamis	(Wiart 2024)
	*Koompassia malaccensis* Maing^†ψ^	Allergy, asthma, bloating, body aches, convulsions, Blood in stools, gastritis, stomachaches, swollen gums, toothaches (Bajau)	raja kayu	(Foo et al. 2016)
	*Millettia nieuwenhuisii* J.J. Smith^†ψ^	Thrush (Murut)	ramus	(Kulip 2003)
Pandaceae Engl. & Gilg (1913)	*Galearia fulva* (Tul.) Miq.^†ψ^	Medicinal food (Dusun)	sanggara	(Voeks and Nyawa 2006)
Phyllanthaceae Martinov (1820)	*Bridelia stipularis* (L.) Bl.	Fever, diabetes, postpartum, thrush (Dusun)	belingkut	(Kulip et al. 2003)
		Fever, diabetes, postpartum, thrush (Kadazan)		(Kulip et al. 2003)
		Diabetes, thrush (Murut)	bolingkut	(Kulip et al. 2003)
**Rosales Bercht. & Presl. (1820)**				
Moraceae Link (1831)	*Ficus retusa* L. ^ψ^	Shivers (Dusun, Kadazan)	hintotobu	(Kulip 1997)
		Medicinal (Murut)	sialbon rindoh	(Wiart 2024)
**Malvales Juss. ex Bercht. & J. Presl (1820)**				
Dipterocarpaceae Blume (1825)	*Shorea parvistipulata* F. Heim^†ψ^	Fatigue (Murut)	roloi	(Kulip 2003)
**Myrtales Juss. ex Bercht. & J. Presl (1820)**				
Melastomataceae Juss. (1789)	*Dissochaeta monticola* Bl.^†ψ^	Blowgun darts poison (Murut)	bina	(Wiart 2024)
	*Melastoma beccarianum* Cogn.^†ψ^	Blemishes (Dusun) ?	duduk abai	(Voeks and Nyawa 2006)
**Sapindales Juss. ex Bercht. & J. Presl (1820)**				
Anacardiaceae R.Br. (1818)	*Mangifera pajang* Kosterm. ^ψ^	Cancer, high cholesterol, medicinal food (Dusun)	bambangan	(Maid et al. 2017)
Burseraceae Kunth (1824)	*Canarium littorale* Bl.^†ψ^	Medicinal food (Dusun)	adal	(Voeks and Nyawa 2006)
	*Dacryodes incurvata* (Engl.) H.J. Lam^†ψ^	Medicinal food (Dusun)	nguluon	(Voeks and Nyawa 2006)
Sapindaceae Juss. (1789)	*Guioa bijuga* (Hiern) Radlk.^†ψ^	Medicinal food (Rungus)	anggil	(Wiart 2024)
	*Nephelium macrophyllum* Radlk.^†ψ^	Medicinal food, magic rituals (Dusun)	mbokot	(Voeks and Nyawa 2 2006)
	*Nephelium uncinatum* Radlk. ex Leenh.^†ψ^	Medicinal food (Dusun)	kamanggis	(Voeks and Nyawa 2 2006)
**[Caryophyllanae Takhtajan (1967)]**				
**Caryophyllales Juss. ex Bercht. & J. Presl (1820)**				
Nepenthaceae Dumort. (1829)	*Nepenthes ampullaria* Jack ^ψ^	Respiratory diseases (Lundayeh)	telungau becuk	
**[Asteranae Takht. (1967)]**				
**Ericales Bercht. & J. Presl (1820)**				
Primulaceae Batsch ex Borkh (1797)	*Embelia dasythyrsa* Miq.^σ ψ^	Fever, medicinal food (Dusun)	sowolikan	(Wiart 2024)
Symplocaceae Desf. (1820)	*Symplocos odoratissima* Choisy ex Zoll. ^ψ^	Fever, malaria (Lundayeh)	lobo	
**Gentianales Juss. ex Bercht. & J. Presl (1820)**				
Asclepiadaceae Borkh (1797)	*Dischidia rafflesiana* Wall.^†ψ^	Cancer, skin diseases (Bajau)	(Foo et al. 2016)	
Rubiaceae Juss. (1789)	*Chassalia chartacea* Craib ^ψ^	Blurred vision (Dusun)	lansi	(Wiart 2024)
	*Hydnophytum formicarum* Jack ^ψ^	Cancer, diabetes, hypertension (Lundayeh)	sarang semut betina	(Wiart 2024)
	*Ixora capillaris* Bremek^†ψ^	Medicinal (Rungus)	tagandap timulu	(Wiart 2024)
	*Neonauclea gigantea* (Valeton) Merr.^†ψ^	Diarrhea, stomach aches, thrush (Dusun)	mahitap	(Wiart 2024)
	*Myrmecodia platytyrea* Becc.	Drunkenness, hypertension, poison antidote (Dusun)	rajah ubat	(Wiart 2024)
		Hypertension (Lundayeh)		(Wiart 2024)
		Diabetes (Murut)	sarang semut	(Wiart 2024)
		Cancer, diabetes, fever, headaches,	sarang semut	(Wiart 2024)
		hypertension, kidney diseases,		
		poison antidote, sinusitis, tuberculosis (Bajau)		
	*Paederia verticillata* Bl.^ψ^	Intestinal worms (Dusun)	taud	(Wiart 2024)
		Intestinal worms (Kadazan)	taud	(Wiart 2024).
	*Praravinia suberosa* (Merr.) Bremek^†ψ^	Medicinal (Murut)	kingkimu	(Kulip 2003)
	*Psychotria gyrulosa* Stapf^†ψ^	Headaches (Dusun)	siroromuk	(Wiart 2024)
	*Rennellia borneensis* Baill.^†ψ^	Medicinal	Sabah ginseng	(Wiart 2024)
**Lamiales Bromhead (1838)**				
Oleaceae Hoffmannsegg et Link (1809)	*Jasminum aculeatum* Blco^†ψ^	Flatulence (Murut)	onsom onsom	(Kulip 2003)
	*Jasminum bifarium* Wall^†ψ^	Sore eyes (Lundayeh)	bunga melor	(Wiart 2024)
**Solanales Juss. ex Bercht. & J. Presl (1820**)				
Convolvulaceae Juss. (1789)	*Merremia gracilis* E.J.F. Campb. & Argent^†ψ^	Asthma, diarrhea, fatigue, jaundice, pancreatitis (Dusun)	malagatas	(Kulip 1997)
		Asthma, diarrhea, fatigue, jaundice, pancreatitis (Kadazan)	malagatas	(Kulip 1997)
	*Merremia peltata* (L.) Merr.	Diarrhea, flatulence, hair loss, babas (Kulip 2003; Kulip)	stomach aches, wounds (Dusun)	2014; Wiart 2024)
		Diarrhea, wounds (Kadazan)		(Kulip 2003)
**Asterales Link (1829)**				
Asteraceae Martinov (1820)	*Crassocephalum crepidioides* (Benth.) S. Moore	Cancer (Murut)	kinsau	(Awang-Kanak and F Foo 2023)
		Ageing, medicinal food (Dusun)	koyundou	(Awang-Kanak and Foo 2023)

^†^ : no cytotoxic study; ψ: no toxicological study.

**Table 2.** Geographical distribution, habitat and cultivability.

[Subclass]					
(Superorder)					
**Order**					
Family	Genus, species, authority	habitat	rarity	cultivability	(references)
**[Lycopodiidae Bek. (1862) (Lycophytes)]**					
Selaginellales Prantl (1854)					
Selaginellaceae Willk. (1854)	*Selaginella argentea* (Wall. ex Hook. & Grev.) Spring^σ^	mossy grounds	not rare	cultivable	(Suranga et al., 2018)
**Ophioglossales Link. (1833)**					
Ophioglossaceae Martinov (1820)	*Helminthostachys zeylanica* (L.) Hook.	Forest	rare	cultivable	(Um, 2010)
**[Polypodiidae Cronquist, Takht. & W. Zimm. (1966)]**					
**Blechnales Pic. Sem. ex Reveal (1993)**					
Blechnaceae Newmann (1844)	Stenochlaena palustris (Burm. f.) Bedd.	near mangroves	not rare	cultivable	(Uda et al., 2020)
Gleicheniales Link (1825)					
Gleicheniaceae C. Presl (1825)	*Gleichenia truncata* (Willd.) Sprain	roadsides	not rare	?	
**Polypodiales Link (1833)**					
Athyriaceae Alston (1956)	*Diplazium cordifolium* Bl.	forest	rare	?	
	*Diplazium esculentum* (Retz.) Sw.	riverbanks	not rare	cultivable	(Singh and Johari, 2018)
Polypodiaceae Link J. Presl & C. Presl (1822)	*Drymoglossum piloselloides* (L.) C. Presl	roadside	not rare	?	
	*Drynaria sparsisora* (Desv.) T. Moore	roadside	not rare	?	
Schizaeales Schimp. (1869)					
Lygodiaceae M. Roem. (1840)	*Lygodium circinnatum* Sw.	edges of forests	not rare	cultivable	(Cunningham and Brinckmann, 2023)
	*Lygodium salicifolium* C. Presl	roadsides	not rare	?	
Nephrolepidaceae Pic. Serm. (1975)	*Nephrolepis acutifolia* (Desv.) Christ	seashores	not rare	?	
Pteridaceae E.D.M. Kirchn. (1831)	*Acrostichum aureum* L.	mangroves	not rare	?	
**[Gnetidae Pax (1894])**					
**Gnetales Blume (1835)**					
Gnetaceae Blume (1833)	*Gnetum macrostachyum* Hook.f.^σµ^	forest	rare	?	
**[Magnoliidae Novák ex Takht. (1967)]**					
**(Austrobaileyanae Doweld ex M.W. Chase & Reveal (2009))**					
**Austrobaileyales Takht. ex Reveal (1992)**					
Schisandraceae Blume (1830)	*Kadsura borneensis* A.C. Sm^β^	mountain	rare	?	
	*Kadsura lanceolata* King^σϕ^	mountain	rare	?	
**(Magnolianae Takht. (1967))**					
**Laurales Juss. ex Bercht. & Presl (1820)**					
Lauraceae Juss. (1789)	*Eusideroxylon zwageri* Teijsm. & Binn.^σ^	forest	rare	cultivable	(Irawan 2012)
	*Litsea garciae* Vidal.^βπ^	forest	rare	cultivable	(Ekamawanti et al., 2023)
**Magnoliales Bromhead (1838)**					
Annonaceae Juss. (1789)	*Artabotrys roseus* Boerl.^β^	forest	rare	?	
	*Goniothalamus roseus* Stapf^β^	forest	rare	?	
	*Goniothalamus velutinus* Airy Shaw^β^	forest	rare	?	
	*Goniothalamus woodii* Merr ^β^	forest	rare	?	
	*Polyalthia tenuipes* Merr.^βπ^	forest	rare	?	
**(Lilianae Takht. (1967))**					
**Commelinales Mirb. ex Bercht. & J. Presl (1820)**					
Eriocaulaceae Martinov (1820)	*Eriocaulon longifolium* Nees ex Kunth	rice paddy	not rare	?	
Poaceae Barnhart (1895)	*Dendrocalamus asper* (Schult. f.) Backer ex K. Heyne	village	not rare	cultivable	(Singh et al., 2004)
	*Garnotia acutigluma* (Steud.) Ohwi	mountain slopes	not rare	?	
	*Panicum palmifolium* J. Koenig	open forest	not rare	?	
Zingiberaceae Martinov (1820)	*Boesenbergia pulchella* (Ridl.) Merr.^β^	forest	rare	cultivable	(Saensouk et al., 2025)
	*Plagiostachys albiflora* Ridl.^σ^	forest	rare	?	
**(Ranunculanae Takht. ex Reveal (1992))**					
**Ranunculales Juss. ex Bercht. & J. Presl (1820)**					
Menispermaceae Juss. (1789)	*Pycnarrhena tumefacta* Miers^σπϕµ^	forest	rare	cultivable	
**(Rosanae Takht. (1967])**					
**Vitales Juss. ex Bercht. & J. Presl (1820)**					
Vitaceae Juss. (1789)	*Ampelocissus polita* (Miq.) Pelser	forest	rare	?	
**Fabales Bromhead (1838)**					
Fabaceae Lindley (1836)	*Airyantha borneensis* (Oliv.) Brummitt^βπ^	forest	rare	?	
	*Koompassia malaccensis* Maing^σ^	forest	rare	?	
	*Millettia nieuwenhuisii* J.J. Smith^β^	forest	rare	?	
Pandaceae Engl. & Gilg (1913)	*Galearia fulva* (Tul.) Miq.	Forest	rare	?	
Phyllanthaceae Martinov (1820)	*Bridelia stipularis* (L.) Bl.	Forest	rare	?	
**Rosales Bercht. & Presl. (1820)**					
Moraceae Link (1831)	*Ficus retusa* L.	gardens	not rare	cultivable	(Adeoluwa et al., 2014)
**Malvales Juss. ex Bercht. & J. Presl (1820)**					
Dipterocarpaceae Blume (1825)	*Shorea parvistipulata* F. Heim^β^	forests	rare	cultivable	(Susanty, 2019)
**Myrtales Juss. ex Bercht. & J. Presl (1820)**					
Melastomataceae Juss. (1789)	*Dissochaeta monticola* Bl.^σ^	forest	rare	?	
	*Melastoma beccarianum* Cogn.^β^	open land	rare	?	
**Sapindales Juss. ex Bercht. & J. Presl (1820)**					
Anacardiaceae R.Br. (1818	*Mangifera pajang* Kosterm.^β^	forest	cultivable		(Tinggal and Tee, 1994)
Burseraceae Kunth (1824)	*Canarium littorale* Bl.^†^	swamp forest	rare	?	
	*Dacryodes incurvata* (Engl.) H.J. Lam^σπ^	forest	rare	?	
Sapindaceae Juss. (1789)	*Guioa bijuga* (Hiern) Radlk.^σπ^	forest	rare	?	
	*Nephelium macrophyllum* Radlk.^β^	forest	rare	?	
	*Nephelium uncinatum* Radlk. ex Leenh.^σ^	forest	rare	cultivable	(Matius et al., 1998)
**(Caryophyllanae Takhtajan (1967])**					
**Caryophyllales Juss. ex Bercht. & J. Presl (1820)**					
Nepenthaceae Dumort. (1829)	*Nepenthes ampullaria* Jack^σπϕµ^	peat swamp forest	rare	cultivable	(Isnaini, and Novitasari, 2023)
**(Asteranae Takht. (1967))**					
**Ericales Bercht. & J. Presl (1820)**					
Primulaceae Batsch ex Borkh (1797)	*Embelia dasythyrsa* Miq.^σ^	forest	rare	?	
Symplocaceae Desf. (1820)	*Symplocos odoratissima* Choisy ex Zoll.^σπϕ^	forest	rare	?	
**Gentianales Juss. ex Bercht. & J. Presl (1820)**					
Asclepiadaceae Borkh (1797)	*Dischidia rafflesiana* Wall.	Swamps	rare	cultivable	(Scott and Sargant, 1893)
Rubiaceae Juss. (1789)	*Chassalia chartacea* Craib	forest	rare	?	
	*Hydnophytum formicarum* Jack	seashores	rare	cultivable	(Huxley, 1978)
	*Ixora capillaris* Bremek^β^	coastal forest	rare	?	
	*Neonauclea gigantea* (Valeton) Merr.^β^	open forest	rare	?	
	*Myrmecodia platytyrea* Becc.	open forest	not rare	cultivable	
	*Paederia verticillata* Bl.^σπϕ^	forest	rare	?	
	*Praravinia suberosa* (Merr.) Bremek^β^	forest	rare	?	
	*Psychotria gyrulosa* Stapf^β^	forest	rare	?	
	*Rennellia borneensis* Baill.^β^	forest	rare	?	
**Lamiales Bromhead (1838)**					
Oleaceae Hoffmannsegg et Link (1809)	*Jasminum aculeatum* Blco^βπϕ^	coastal forest	rare	?	
	*Jasminum bifarium* Wall	forest	rare	?	
**Solanales Juss. ex Bercht. & J. Presl (1820**)					
Convolvulaceae Juss. (1789)	*Merremia gracilis* E.J.F. Campb. & Argent	forest	rare	?	
	*Merremia peltata* (L.) Merr.	Forest	rare	cultivable	(Kirkham, 2004)
Asteraceae Martinov (1820)	*Crassocephalum crepidioides* (Benth.) S. Moore	roadsides	not rare	cultivable	(Dossou et al., 2019)

β: Endemic solely in Borneo; σ: Sundaland; ϕ: Wallacea; π: Philippines; µ: Sahuland.

### **Selaginella argentea** (wall. ex-hook. & grev.) spring (Selaginellaceae)

**Figure 1.**
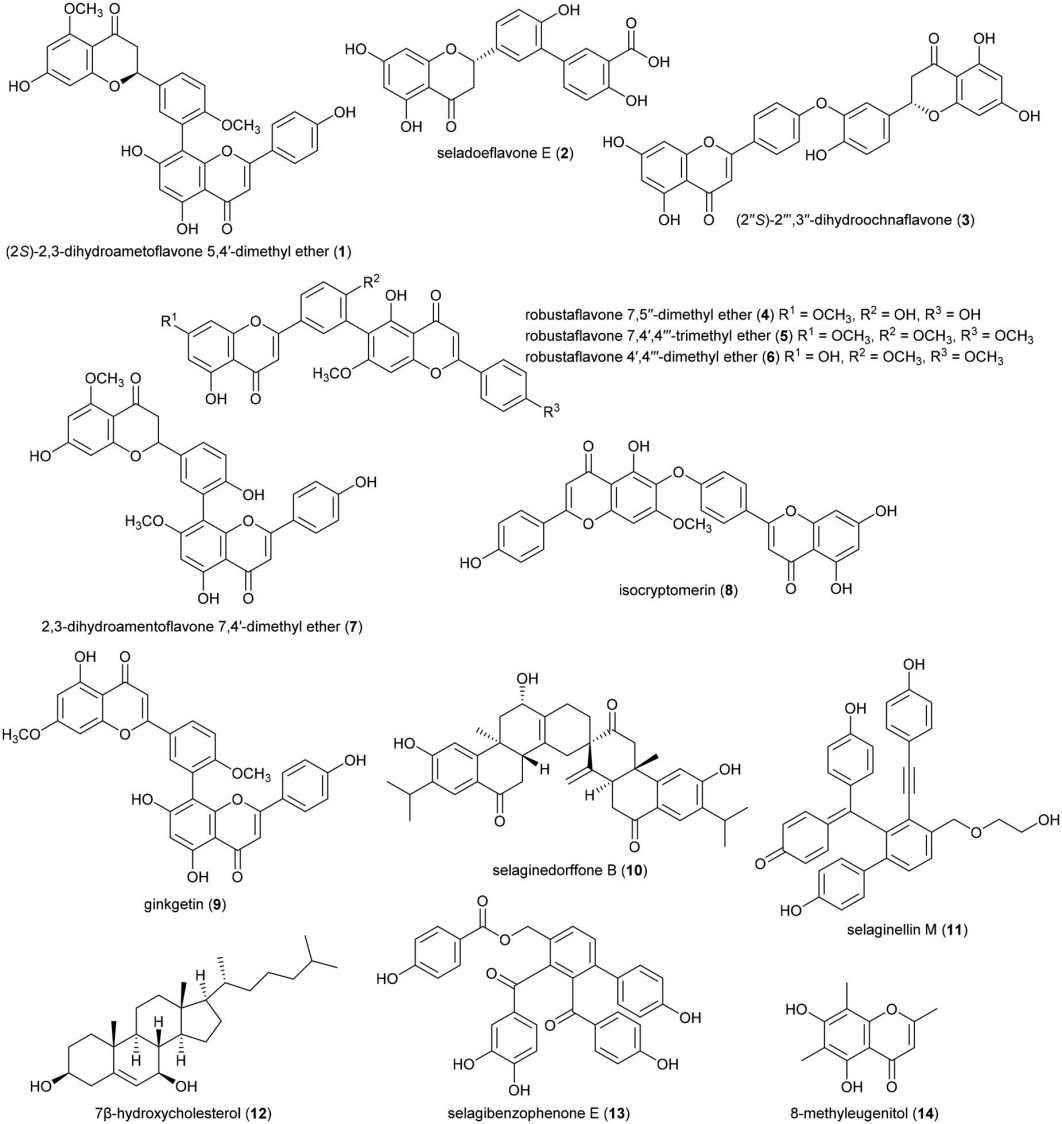
Cytotoxic natural products of plants of the genus *Selaginella.*

Plants in the genus *Selaginella* P. Beauv. (1804) produce cytotoxic phenolic principles (Figure 1). We can cite the biflavone (2*S*)-2,3-dihydroametoflavone 5,4′-dimethyl ether (**1**) and flavanone seladoeflavone E (**2**) (K562; IC_50_: 8.1 µM) from *Selaginella doederleinii* Hieron (Zou et al. 2017; Liu et al. 2021). Other examples are (2″*S*)-2″,3″-dihydroochnaflavone (**3**) and robustaflavone 7,5′′-dimethyl ether (**4**) from *Selaginella trichoclada* Halston active against the breast cancer cell line (MCF-7) with IC_50_ values of 7.7 and 6.9 μM, respectively (Xie et al. 2022). From the aerial parts of *Selaginella delicatula* (Desv.) Alston (collected in Taiwan), robustaflavone 7,4′,4′′′-trimethyl ether (**5**), robustaflavone 4′,4″′-dimethyl ether (**6**), and 2,3-dihydroamentoflavone 7,4′-dimethyl ether (**7**) were found to be cytotoxic to murine lymphocytic leukemia cell line (P388) with IC_50_ values of 4.7, 1.4, and 3.5 µg/mL, repectively (Chen et al. 2005). We can also cite isocryptomerin (**8**) from the leaves of *Selaginella willdenowii* (Desv.) Baker (collected in Panama), (ZR-75-1; IC_50_: 0.5 µg/mL) (Silva et al. 1995), some chalcone-flavanone biflavonoids from *Selaginella trichoclada* Alston (Xie et al. 2022), and ginkgetin (**9**) from *Selaginella moellendorffii* Hieron (OVCAR-3: IC_50_:1.8 µg/mL) (Sun et al. 1997).

Other cytotoxic principles in this genus are abietane-type diterpenes (Wu et al. 2020) such as selaginedorffone B (**10**) from *S. moellendorffii* (MCF-7; IC_50_: 9 μM) (Ke et al. 2018), phenolic compounds as in selaginellin M (**11**) (HeLa; IC_50_: 28.5 μM) (Yang et al. 2012), 7β-hydroxycholesterol (**12**) (HT-115; IC_50_: 2.7 μg/mL) (Roh et al. 2010), selagibenzophenone E (**13**) (a benzophenone) (SMCC-7721; IC_50_: 15.8 µM) (Long et al. 2023), 8-methyleugenitol (**14**) (a chromone) from *Selaginella siamensis* Hieron. (HuCCA-1; IC_50_: 20.8 µM) (Thamnarak et al. 2022), and cyclopeptides (Yan et al. 2022). To date, no phytochemical, pharmacological, or toxicological studies appear to have been conducted on *S. argentea*. This plant is cultivable (Table 2).

### **Helminthostachys zeylanica** (L.) hook. (Ophioglossaceae)

**Figure 2.**
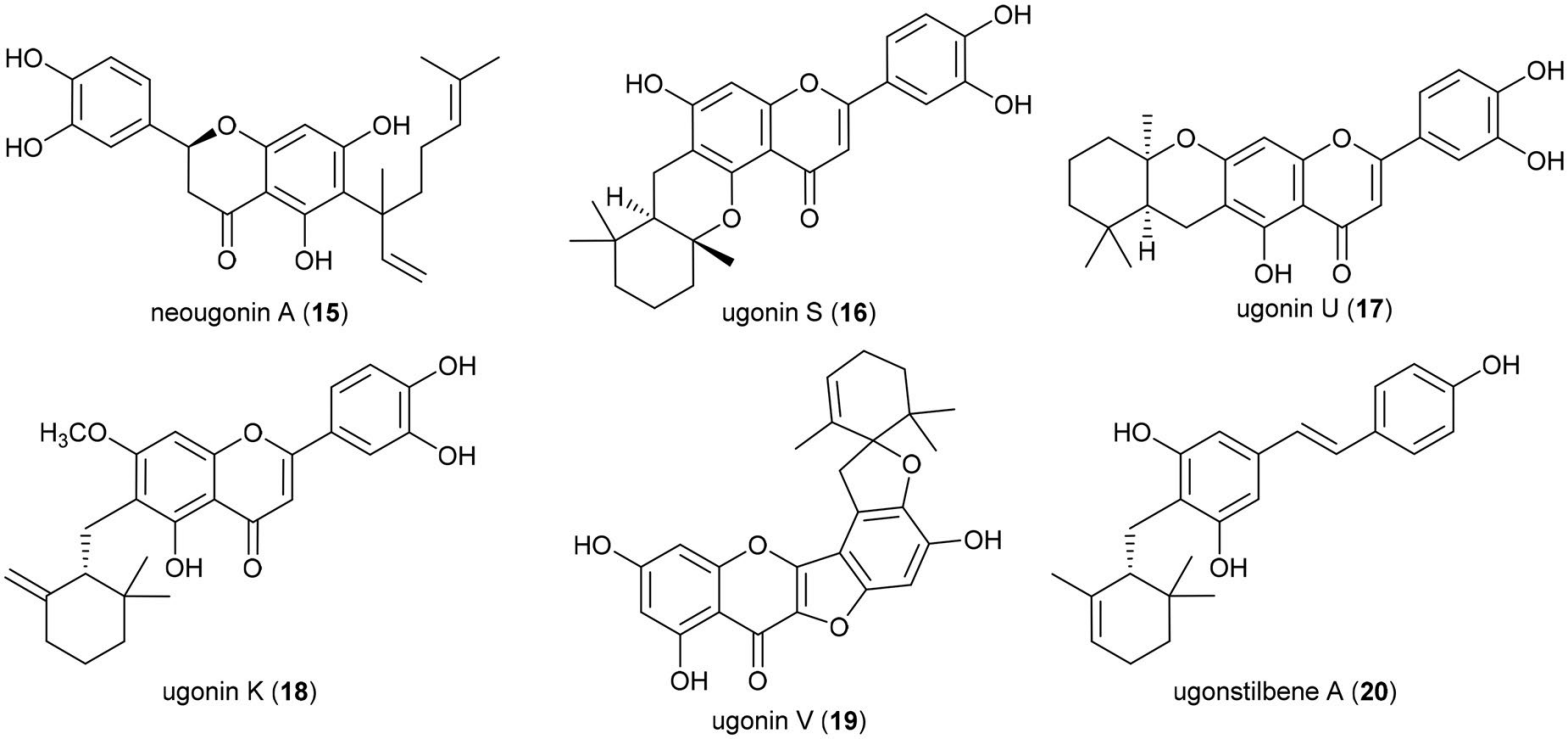
Cytotoxic natural products of *H. zeylanica.*

Anticancer properties have been demonstrated *in vitro* and involve a series of cytotoxic and anti-inflammatory prenylated flavonoids, rare monoterpene-flavonoid meroterpenes (Figure 2). An ethyl acetate fraction, at the concentration of 80 µg/mL, induced the apoptosis of gastric adenocarcinoma cell line (AGS) with cleavage of poly (ADP-ribose) polymerase, decreased expression of Bcl-2 and cyclooxygenase 2 (Tsai et al. 2021). The plant produces anti-inflammatory prenylated flavanones such as neougonin A (**15**), which inhibited lipopolysaccharide-induced nitric oxide production in mouse monocyte macrophages (RAW264.7) cell line, with an IC_50_ value of 3.3 µM (Cao et al. 2016).

Other principles are prenylated flavones such as ugonin S (**16**) and U (**17**) (Huang et al. 2009; Su et al. 2016), cytotoxic to acute lymphoblastic leukemia cell line (CEM) and lung cancer cell line (H460) (Lin et al. 2025). Ugonin K (**18**) inhibited the growth of basal cell carcinoma cell line *via* reduction of mitochondrial membrane potential, the expression of p53, and subsequent activation of caspases-8, −9, and −3 leading to apoptosis (Chan et al. 2013). Ugonin V (**19**) given intraperitoneally thrice weekly for 4 weeks at a dose of 15 mg/kg inhibited metastasis of chondrosarcoma tumors in lungs of mice, *via* at the tumor cellular level and inhibition of cathepsin V expression (Tran et al. 2025). Other cytotoxic principles in this fern are prenylated stilbenes such as ugonstilbene A (**20**) (Lin et al. 2023). This fern is used as a medicinal food by the Dusun and as a remedy for cancer by the Lundayeh (Table 1). In a clinical study, patients receiving rhizome powder (1 g three times daily for 42 days) experienced no adverse effects. (Su et al. 2022). This fern is cultivable (Table 2). Clinical trials are needed to confirm its efficacy and safety.

#### **Stenochlaena palustris** (burm. f.) bedd. (Blechnaceae)

**Figure 3.**
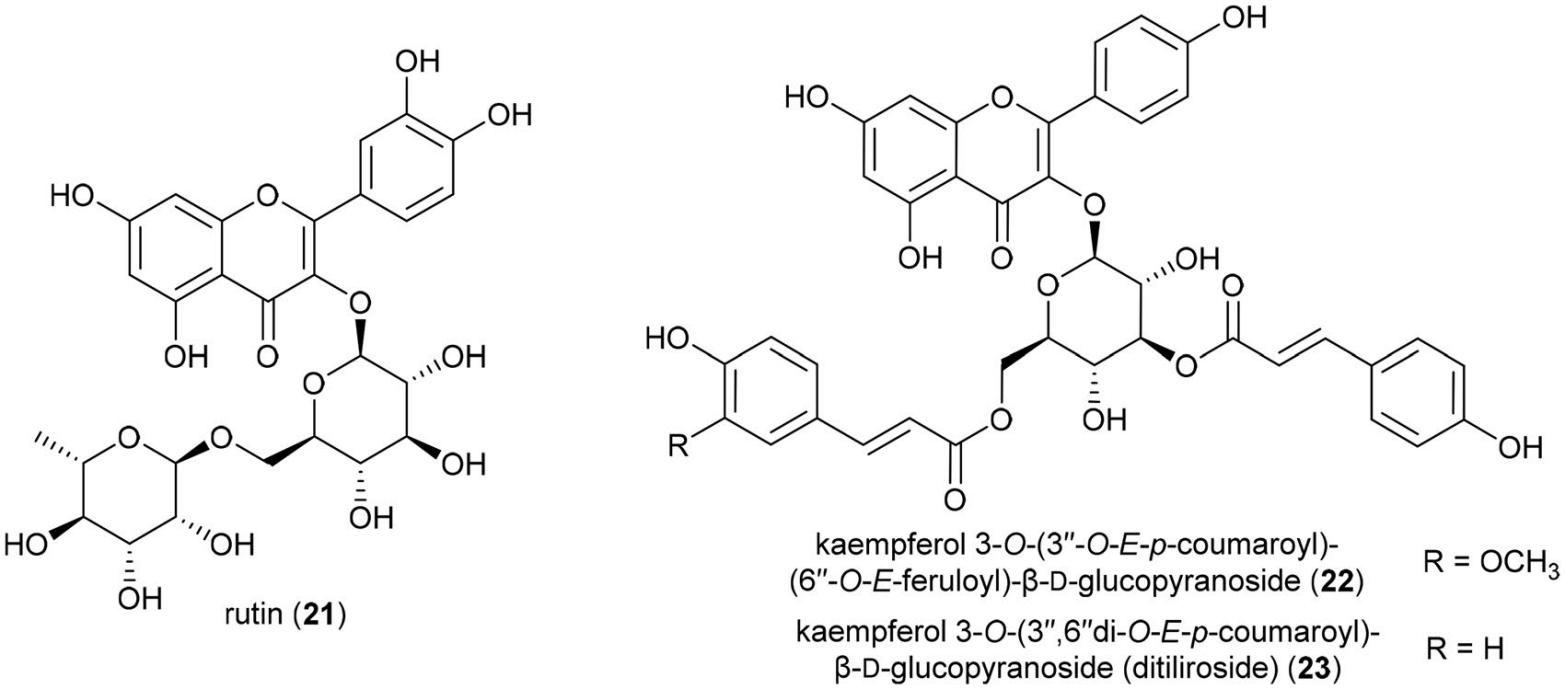
Cytotoxic flavonoids of *S. palustris.*

The Dusun use this fern as a medicinal food (Wiart, 2024) (Table 1). The ethanolic extract of its leaves (harvested in Malaysia) has been shown to be toxic to cervical cancer cell line (HeLa) (IC_50_: 5.8 µg/mL) (Arullappan et al. 2017). This fern produces flavone glycosides, including rutin (**21**), as well as acylated flavone glycosides (Hendra et al. 2024) (Figure 3). Rutin (**21**) has been shown to be moderately cytotoxic to malignant melanoma cell line (RPMI-7951) and (SK-MEL-28), with IC_50_ values of 64.4 and 47.4 µM, respectively (Pinzaru et al. 2021). Kaempferol 3-*O*-(3″-*O*-E-p-coumaroyl)-(6″-*O*-E-feruloyl)-β-D-glucopyranoside (**22**) and kaempferol 3-*O*-(3″,6″di-*O*-E-p-coumaroyl)-β-D-glucopyranoside (or ditiliroside) (**23**) from this fern inhibited the proliferation of breast cancer cell line (MDA-MB-231) with IC_50_ values of 70 and 21 µM, respectively (Chear et al. 2019). The toxicity of *S. palustris* remains unstudied. It is cultivable (Table 2).

### **Drynaria sparsisora** (desv.) T. Moor

**Figure 4.**
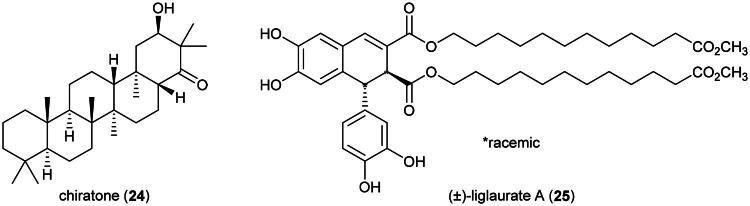
Cytotoxic natural products of plants of the genus *Drynaria.*

Aqueous extract of *Drynaria quercifolia* (L.) J.Sm. and *Drynaria fortunei* (Kunze ex Mett.) J.Sm. were found to be cytotoxic to brine-shrimp s (LC_50_: 7.6 µg/mL) (Runa et al. 2013) and weakly active against MDA-MB-231 cell line (IC_50_ ≈ 500 µg/mL) (Telang et al. 2025). Methanol extract of *D. quercifolia* (L.) J.Sm. inhibited the growth of hepatocellular carcinoma (HepG2) cell line (IC_50_ ≈ 200 µg/mL) (Prasanna et al. 2019). A dichloromethane extract of *Drynaria rigidula* (Sw.) Bedd. was found to be cytotoxic to MCF-7 cell line (IC_50_: 18 µg/mL) (Nugraha et al. 2019). From this fern, a chiratane-type triterpene, chiratone (**24**), was found to inhibit the growth of prostate cancer cell line (PC3) (IC_50_: 1 μM) (Liang et al. 2010) (Figure 4). Isolated from this fern’s rhizome, the long-chain alkylated lignan (+)-liglaurate A (**25**) abrogated the survival of cervical cancer cell line (HeLa) (IC_50_: 0.1 μM) (Wufuer et al. 2022). In Sulawesi, a plant in the genus *Drynaria* (Bory) J. Sm. (1841) has been used to treat cancer (Nurrani et al. 2014). To date, no phytochemical, pharmacological, or toxicological studies appear to have been conducted on *D. sparsisora*.

### **Lygodium circinnatum** Sw. (Lygodiaceae)

There are a number of studies that demonstrate the cytotoxic activity of organic extracts from ferns of the genus *Lygodium* Sw. (1801): *Lygodium venustum* Sw. (ethanol leaves; NCTC929; IC_50_: 500 µg/mL) (Morais-Braga et al. 2013), *Lygodium microphyllum* (Cav.) R. Br. (ethyl acetate; P388; IC_50_: 50.1 µg/mL) (Kuncoro 2017) and *Lygodium flexuosum* (L.) Sw. (hexane; Hep3B; IC_50_: 32 µg/mL). In the latter case, apoptosis was observed with cleavage of the poly(ADP-ribose) polymerase (Wills and Asha 2009).

**Figure 5.**
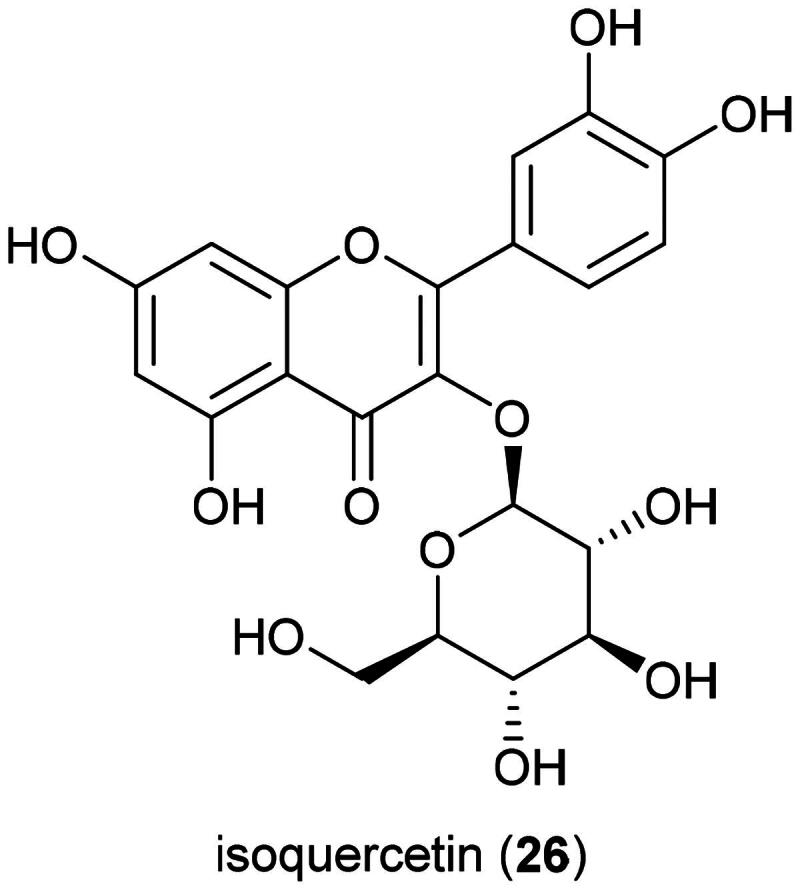
Cytotoxic flavonol glycoside of plants of the genus *Lygodium.*

Plants in this genus produce ecdysteroids and their glycosides (Guo-Gang et al. 2012), and flavonol glycosides, including isoquercetin (**26**) (Kuncoro et al. 2017) (Figure 5). Isoquercetin (**26**) (Figure 5), given orally to mice, xenografted with colorectal adenocarcinoma cell line (HT-29), at a dose of 17 µg/g over a week, caused a decrease in tumor volume (da Silva et al. 2022). Other principles in these ferns are naphthoquinones (Chen et al. 2010), triterpenes (Han et al. 2012), phenylpropanoid glycosides (Duan et al. 2012), and diterpenes (Yamauchi et al. 1996). To date, no phytochemical, pharmacological, or toxicological studies appear to have been conducted on *L. circinnatum.* This fern is cultivable (Table 2).

### **Litsea garciae** vidal

**Figure 6.**
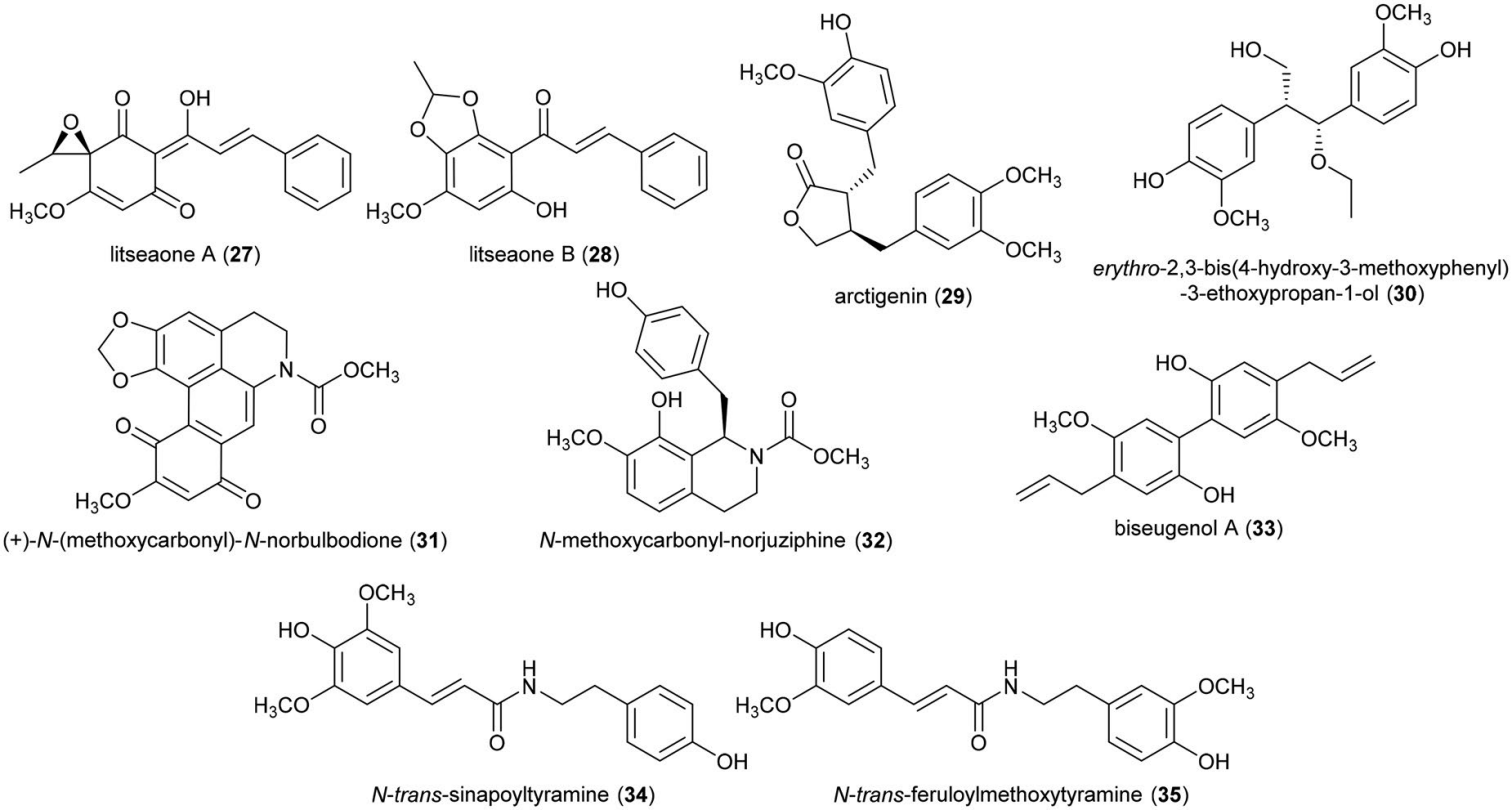
Cytotoxic natural products of plants of the genus *Litsea.*

A methanolic extract of this plant has been shown to be cytotoxic against various cancer cell lines (Kutoi et al. 2012), but to date, its active principles remain unknown. Plants of the genus *Litsea* Lam (1792) produce different types of natural cytotoxic substances such as litseaone A (**27**) and B (**28**) (chalcones) (Kageji et al. 2018; Kageji et al. 2019) from *Litsea rubescens* Lecomte (HL-60; IC_50_: of 6.1 and 10.2 µg/mL, respectively) (Li et al. 2011) and from *Litsea cubeba* (Lour.) Pers, arctigenin (**29**) (a dibenzylbutyrolactone lignan), *erythro*-2,3-bis(4-hydroxy-3-methoxyphenyl)-3-ethoxypropan-1-ol (**30**) (a stilbene) (Guo et al. 2015; Li et al. 2019), (*+*)*-N*-(methoxycarbonyl)-*N*-norbulbodione (**31**) (an aporphine alkaloid) (HepG2; IC_50_: 9.5 µM) (Zhang et al. 2012), and *N*-methoxycarbonyl-norjuziphine (**32**) (benzylisoquinoline alkaloid) (MCF-7; IC_50_:15 μM) (Tang et al. 2017) (Figure 6).

Other examples of cytotoxic natural products isolated from in this genus are biseugenol A (**33**) (a phenylpropanoid dimer) from *Litsea costalis* (Nees) Kosterm. (collected in Malaysia) (HepG2; IC_50_: 18 μM) (Hosseinzadeh et al. 2013), *N*-*trans*-sinapoyltyramine (**34**) and *N*-*trans*-feruloylmethoxytyramine (**35**) (amide alkaloids) from *Litsea acuminata* (Blume) Kurata (collected in China) (HeLa) (Tanaka et al. 2009), butanolides (Cheng et al. 2001), and sesquiterpene glycosides (Wang et al. 2018).

Given the numerous cytotoxic natural products in this genus and considering that the Dusun use the edible fruits of *L. garciae* (Hassan et al. 2013) as a medicinal food (Maid et al. 2017) (Table 1), further studies are warranted. This fruit tree is only found in Borneo, the Philippines, and Taiwan (Table 2). The observation of the limited geographical distribution of this plant could lead one to wonder if its distribution might be an arboreal vestige of an Austronesian population, which would confirm the hypothesis of a Taiwanese origin of the Austronesian peoples (Solheim 1984).

### **Artabotrys roseus** boerl. (Annonaceae)

**Figure 7.**
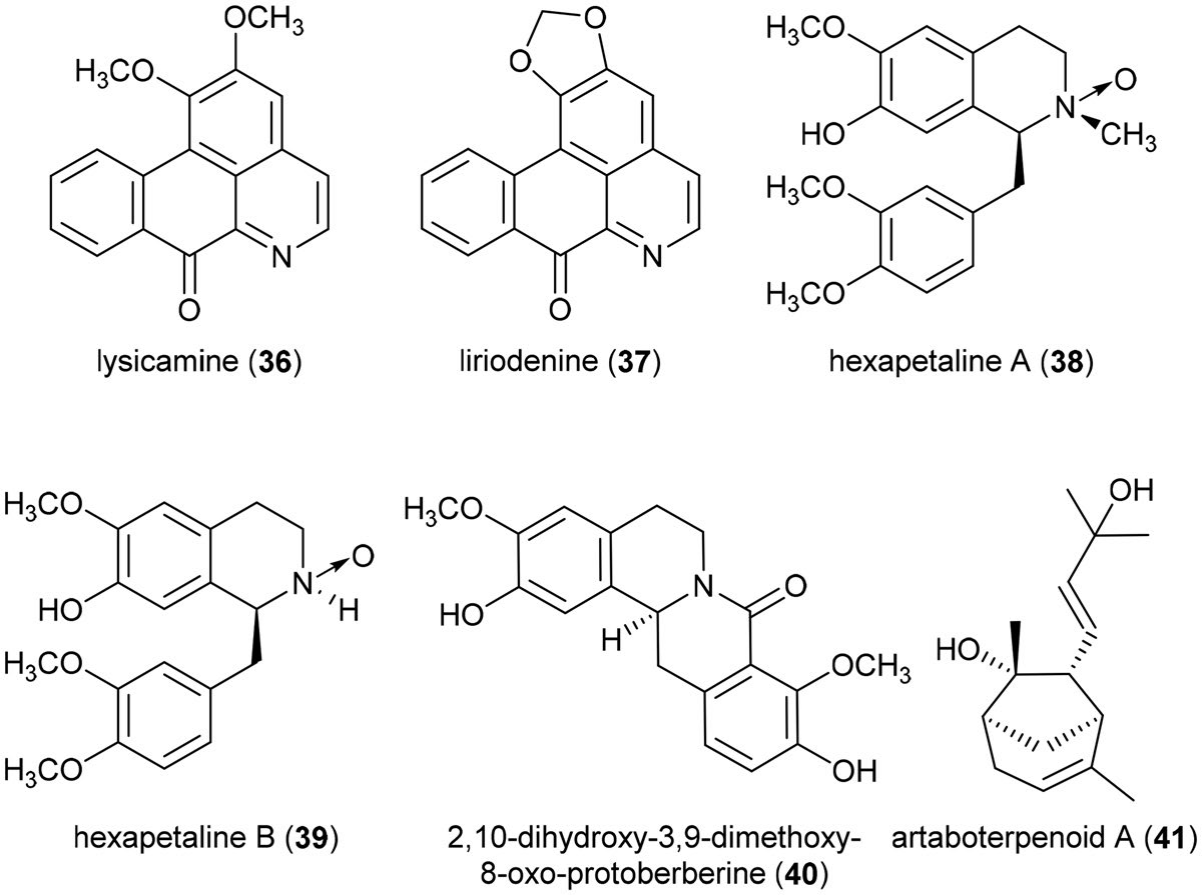
Cytotoxic natural products of plants of the genus *Litsea.*

Plants in the genus *Artabotrys* R.Br. (1820) produce cytotoxic aporphine alkaloids. Examples are lysicamine (**36**), isolated from *Artabotrys crassifolius* Hook. f. & Thomson (collected in Malaysia), toxic to MCF-7 cell line (IC_50_: 3.9 µg/mL) (Kwan et al. 2016), and liriodenine (**37**) (Zhao et al. 2024). Other examples of cytotoxic alkaloids include hexapetalines A (**38**) and B (**39**) (benzylisoquinolines) (Zhou et al. 2015) and the protoberberine alkaloid 2,10-dihydroxy-3,9-dimethoxy-8-oxo-protoberberine (**40**) (Nguemdjo Chimeze et al. 2022) (Figure 7). Plants in this genus also produce cytotoxic sesquiterpenes, such as artaboterpenoid A (**41**) (Xi et al. 2016). The precise medical use of *A. roseus*, employed by both Dusun and Kadazan (Table 1), is unknown. Phytochemical, pharmacological, or toxicological reports on *A. roseus*, endemic to Borneo, are nonexistent.

### **Boesenbergia pulchella** (ridl.) merr. (Zingiberaceae)

**Figure 8.**
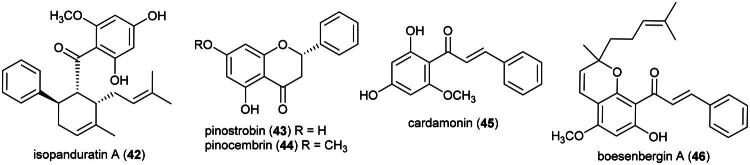
Cytotoxic phenolic compounds of plants of the genus *Boesenbergia.*

Ethanol extract of rhizomes was found to be cytotoxic to MCF-7 cell line (IC_50_: 93 µg/mL) (Jing et al. 2010). Active cytotoxic principles are not elucidated yet, but available evidence suggest phenolic principles. Ethanol and ethyl acetate extracts of rhizomes of *Boesenbergia rotunda* (L.) Mansf. were found to be cytotoxic to HeLa cell line (IC_50_: 56 μg/mL) (Listyawati et al. 2016) and small duct intrahepatic cholangiocarcinoma cell line (RMCCA-1) (IC_50_: 22.6 µg/mL), respectively (Sopitthummakhun et al. 2021). In a subsequent study, an ethyl acetate extract of rhizomes of *B. rotunda* was found to be cytotoxic to A549 cell line (IC_50_: 22.5 µg/mL) on account of isopanduratin A (**42**) (a chalcone derivative), pinostrobin (**43**) and pinocembrin (**44**) (flavanones), and cardamonin (**45**) (a chalcone) which inhibited the growth of human lung cancer cell line (A549) with IC_50_ values of 10.1, 25.3, 36.1, and 5.2 µg/mL, respectively (Han et al. 2023) (Figure 8). Cardamonin (**45**) induced apoptosis in nasopharyngeal carcinoma cell line (HK1) (IC_50_: 27 μg/mL) *via* the activation of caspases-3 and −8, as well as alterations in mitochondrial membrane potential (Break et al. 2021). The prenylated chalcone boesenbergin A (**46**) induced apoptosis in A549 cell line with mitochondrial membrane potential alteration (Isa et al. 2013), as well as with human T4-lymphoblastoid cell line (CEM-SS) (IC_50_: 8 μg/mL) (Ng et al. 2013). A hexane extract of the stems of *Boesenbergia violacea* (K.Larsen & Triboun) Mood & L.M.Prince inhibited the growth of malignant melanoma cell line (A375) (IC_50_: 31.4 µg/mL) (Choosub and Samosorn 2021). The pharmacological properties and any possible toxicities of *B. pulchella*, endemic to Borneo, are currently unknown.

#### **Etlingera elatior** *(jack) R.M. Sm*

Organic extracts demonstrated cytotoxic, antimutagenic, immunostimulant, and anti-inflammatory activities. Ethyl acetate extract of rhizome was found to be cytotoxic for CEM-SS and MCF-7 cell line with IC_50_ values of 4 and 6.2 µg/mL, respectively (Habsah et al. 2005). An ethanol extract of flowers inhibited the growth of MCF-7 and MDA-MB-231 cell line with the IC_50_ of 173.1 and 196.2 μg/mL, respectively (Ghasemzadeh et al. 2015). An essential oil of the leaves was found to inhibit the growth of mouse melanoma cell line (B16F10) (IC_50_: 214.8 μg/mL) (Sangthong et al. 2022). A dichloromethane extract (200 µg/mL) protected Raji cell line against mutations caused by 12-*O*-tetradecanoylphorbol-13-acetate (Habsah et al. 2005). Methanol extract induced the proliferation of human peripheral lymphocytes (Safriani et al. 2021). Furthermore, an ethanol extract of flowers administered intraperitoneally to mice at a dose of 100 mg/kg induced a decrease in cyclooxygenase 2 expression after 7 days of treatment (Syaify et al. 2024). The flowers of this ginger are consumed to maintain good health among the Dusun and could represent an interesting material for the development of onco-preventive nutraceuticals. Although the toxicity profile of these flowers in humans has not been established, oral administration of a flower extract at a single dose of 600 mg/kg in rats did not cause any lethality (Sholihah et al. 2025). Further studies are needed on this common garden plant (Table 2).

### **Pycnarrhena tumefacta** miers (Menispermaceae)

**Figure 9.**
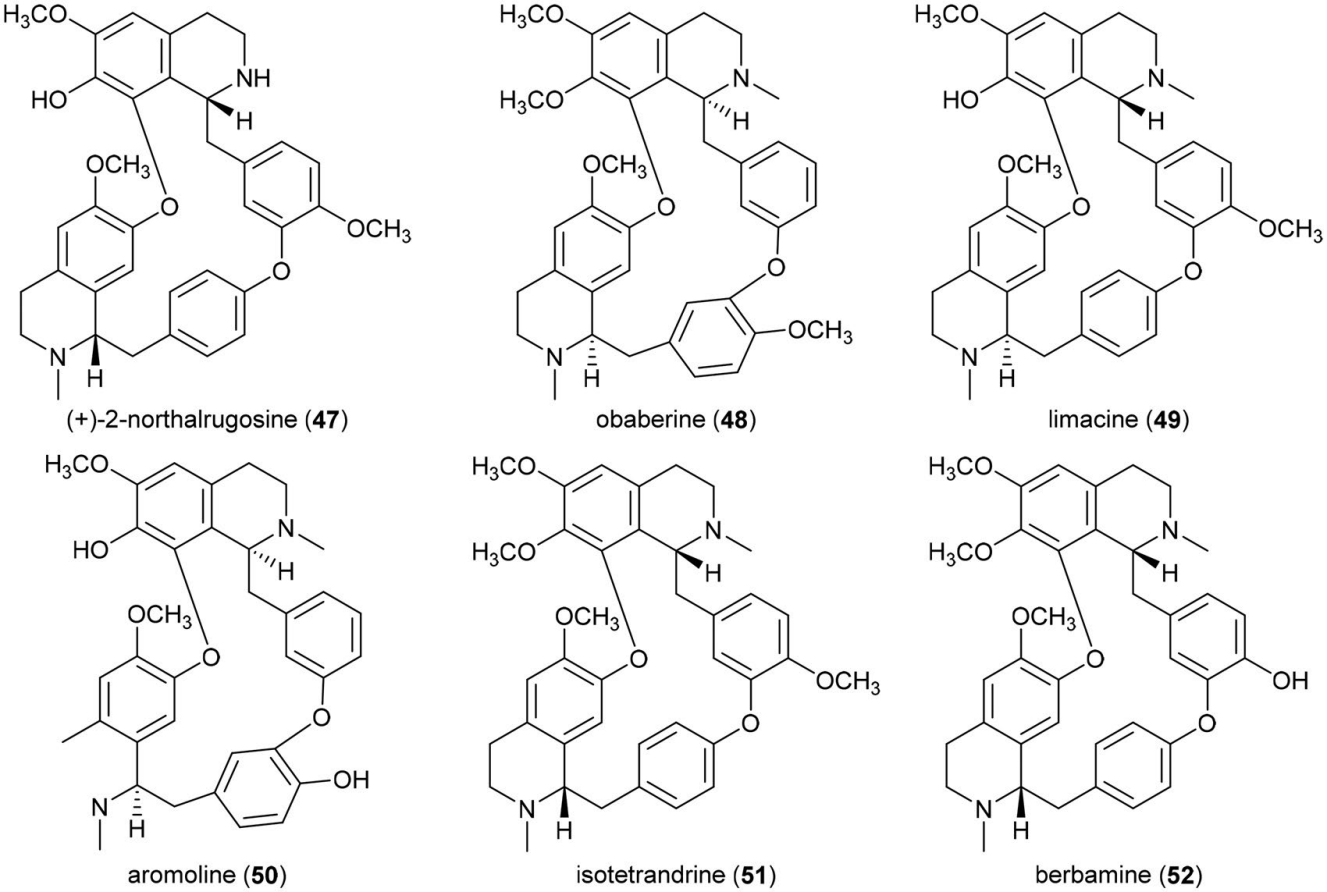
Cytotoxic natural products of plants of the genus of plants of the genus *Pycnarrhena.*

*P. tumefacta* is used as a medicinal food by the Dusun, Kadazan, and Murut (Table 1), and is also consumed locally in Sarawak where it is known as “*sengkubak*” and used as a condiment to impart a salty flavor to dishes (Yusli et al. 2023). An extract from this vine has been shown to be toxic to cervical cancer cell line (HeLa) (Fernandez 2009). An alkaloid extract of the roots of *Pycnarrhena. cauliflora* (Miers) Diels induced apoptosis in ductal carcinoma of the breast cell line (T-47D) (Masriani et al. 2014). Dichloromethane extract of stems (collected in Indonesia) was found to be cytotoxic to T-47D cell line (IC_50_: 59.2 µg/mL) and induced apoptosis (Muharini and Enawaty, 2019). Plants in the genus *Pycnarrhena* Miers ex Hook. f. & Thomson (1855) produce cytotoxic bisbenzylisoquinoline alkaloids such as (+)-2-northalrugosine (**47**) (Abouchacra et al. 1987) from *Pycnarrhena ozantha* Diels (KB; IC_50_: 10.4 µM) (Angerhoferet al. 1999), obaberine (**48**), limacine (**49**), aromoline (**50**), and isotetrandrine (**51**) (Van Beek et al. 1982) from *Pycnarrhena longifolia* (Decne. ex Miq.) Becc. (KB cell line) (Angerhoferet al. 1999), and isotetrandrine (**51**) and berbamine (**52**) from *Pycnarrhena manillensis* S. Vidal (KB cell line) (Bruchhausen et al. 1960; Angerhofer et al. 1999) (Figure 9). More experiments are needed.

### ***Bridelia stipularis*** (L.) Bl. (Phyllanthaceae)

**Figure 10.**
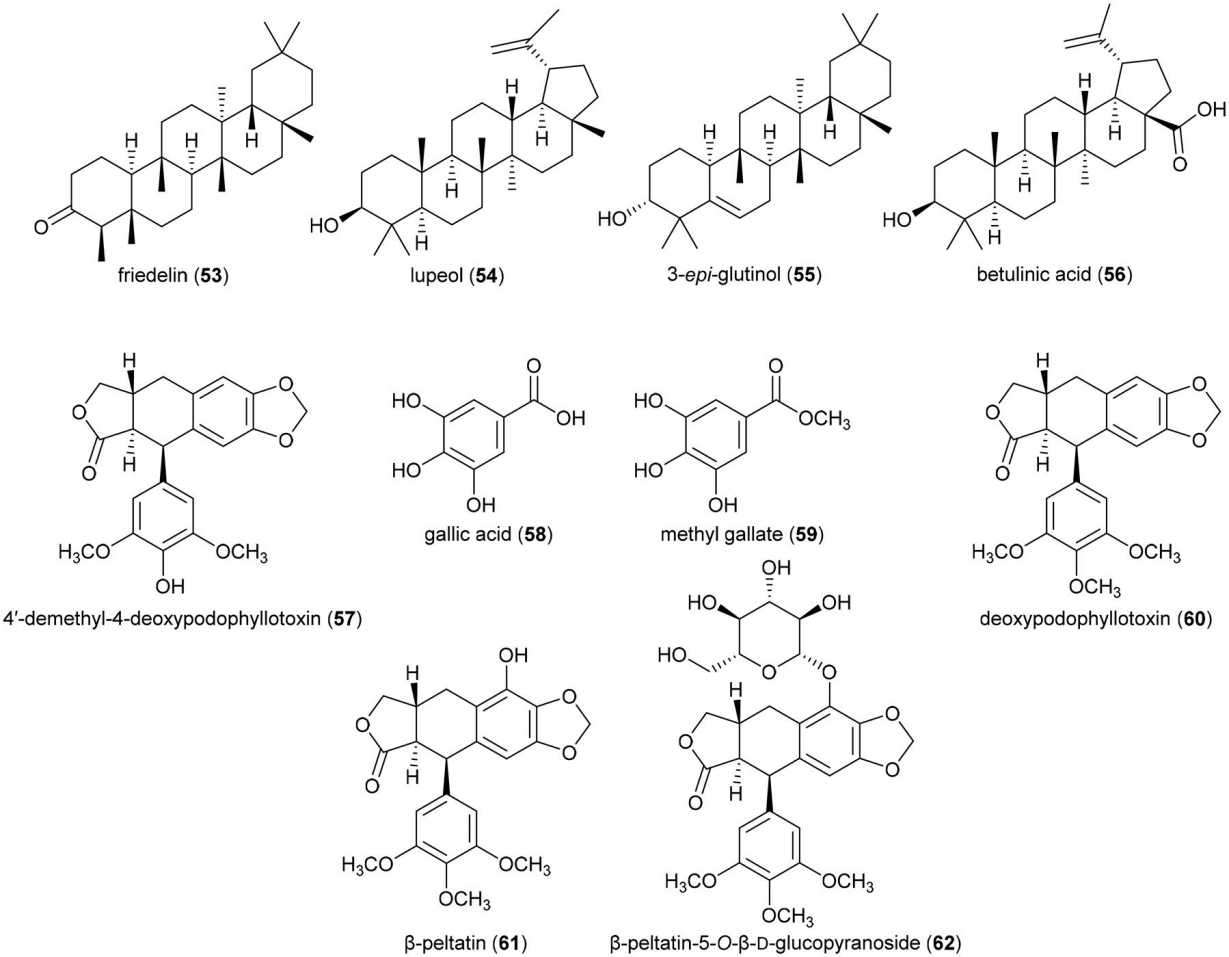
Cytotoxic natural products of plants of the genus *Bridelia.*

This tree produces friedelane-type triterpenes such as friedelin (**53**) and the lupane-type triterpene lupeol (**54**), and a phenolic compound identified as 4-(1,5-dimethyl-3-oxo-4-hexenyl)benzoic acid (Limtragool et al. 2024) (Figure 10). Friedelin (**53**) was found to be cytotoxic to MCF-7 cell line (IC_50_: 1.8 µM) and induced an increase in cellular reactive oxygen species, DNA damage, and apoptosis (Subash-Babu et al. 2017). Lupeol (**54**) inhibited the survival of glioblastoma cell line (U87MG; IC_50_: 13.6 μM) (Nyaboke et al. 2018). 3-*Epi*-glutinol (**55**) (a friedelane-type triterpene) and betulinic acid (**56**) (a lupane-type triterpene) from *Bridelia cambodiana* Gagnep were toxic to HL-60 cell line with IC_50_ values of 5.6 and 6.9 µg/mL, respectively (Khiev et al. 2009). Regarding the biological activity of triterpenes, caution is advised as it depends on their purity level (Jaki et al. 2008).

Plants in the genus *Bridelia* Willd. (1806) have the property of producing aryltetralin lignans of the podophyllotoxin type. This type of lignan constitutes a well-known class of cytotoxic agents. Etoposide (still widely used and forming the basis of several combination chemotherapy protocols) and Teniposide (rarely used today, mainly in the treatment of childhood acute lymphoblastic leukemia) are approved drugs. 4′-Demethyl-4-deoxypodophyllotoxin (**57**), gallic acid (**58**), and methyl gallate (**59**) (benzoic acid derivatives) isolated from the roots of *Bridelia balansae* Tutcher inhibited the growth of HCT-116 cell line with IC_50_ values of 0.02, 20, and 16.3 µM, respectively (Zhao et al. 2023). *Bridelia ferruginea* Benth. produces deoxypodophyllotoxin (**60**) and β-peltatin (**61**) (Pettit et al. 2016). Deoxypodophyllotoxin (**60**) was found to be cytotoxic to A549 and B16F10 with IC_50_ values of 6 and 6.8 ng/mL, respectively. Given intraperitoneally at a dose of 20 mg/kg/day for 14 days to mice xenografted with Lewis lung carcinoma cell line, deoxypodophyllotoxin (**60**) caused a decrease in tumor growth (Kim et al. 2002). β-Peltatin (**61**) induced apoptosis in pancreatic cancer cell line (MIA PaCa-2; IC_50_: 2.5 nM), with cleavage of poly (ADP-ribose) polymerase, activation of caspase-3, and increased expression of cyclin B1. Given intraperitoneally at a dose of 15 mg/kg/day for 40 days to mice xenografted with prostatic adenocarcinoma cell line (PC-3), β-peltatin (**61**) reduced the volume of tumors and increased lifespan (Wu et al. 2023). β-Peltatin-5-*O*-β-D-glucopyranoside (**62**) was found to be cytotoxic to A2780 cell line (IC_50_: 4.9 μM) (Liu et al. 2015). Ethanol extract of leaves of *B. stipularis* given orally to mice at a dose of 500 mg/kg for 14 days to rodents did not cause liver toxicity (Acharyya et al. 2022).

### **Ficus retusa** L. (Moraceae)

**Figure 11.**
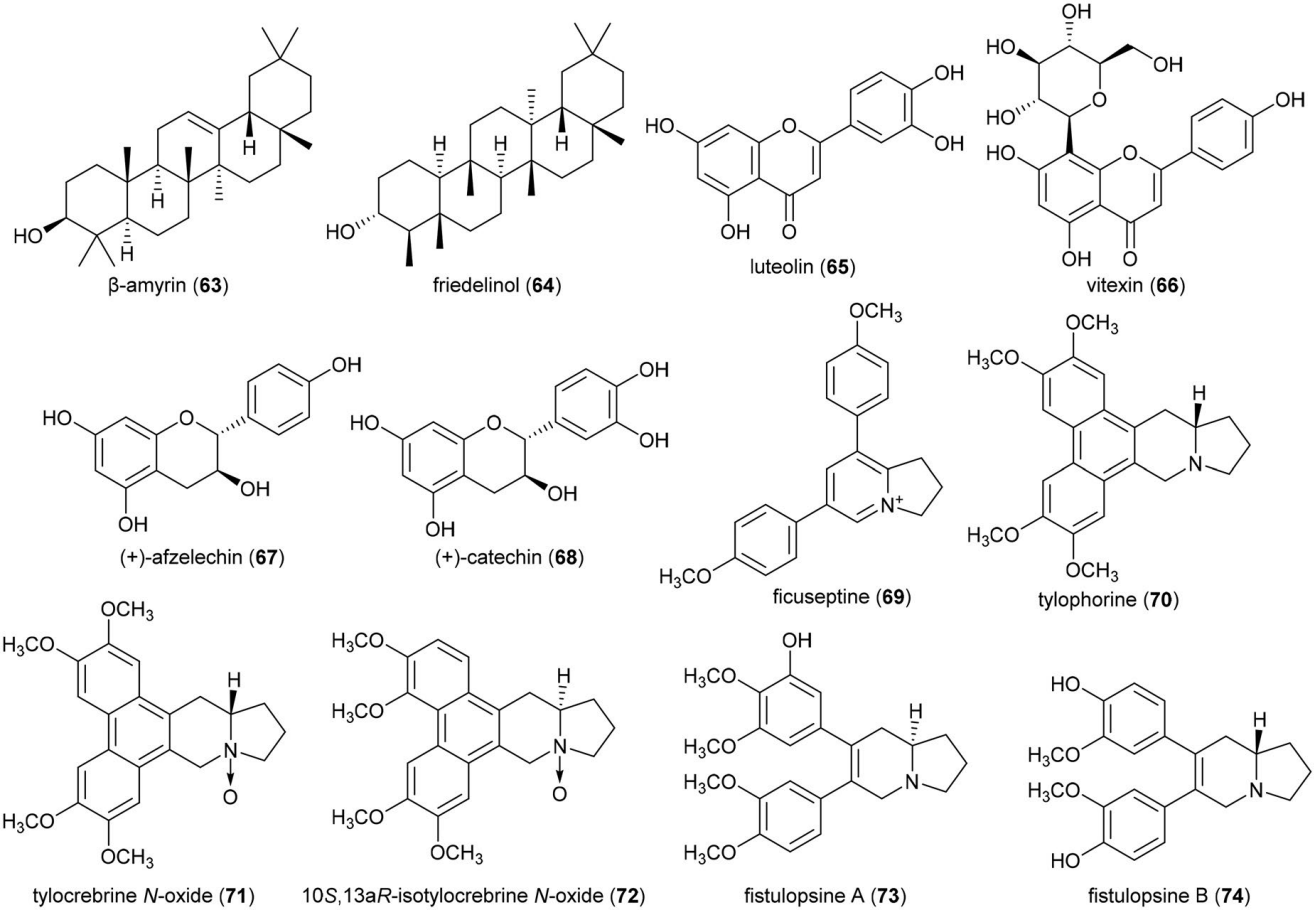
Cytotoxic natural products of plants of the genus *Ficus.*

Ethyl acetate extract of *F. retusa* was found to be cytotoxic to HepG2 cell line (IC_50_: 68.4 µg/mL). The plant produces the oleanane-type triterpene β-amyrin (**63**) (Caco-2; IC_50_: 81 µg/mL) (Maiyo et al. 2016), the friedelane-type triterpene friedelinol (**64**) (THP-1) (Gonçalves Pereira et al. 2020), the flavone luteolin (**65**) (NCI-H460) (Kyoung-Ah et al. 2006), vitexin (**66**) (a flavone C-glycoside), the flavanes (+)-afzelechin (**67**) and (+)-catechin (**68**) (Sarg et al. 2011) (Figure 11). Vitexin (**66**) induced the apoptosis of HCT-116 cell line (IC_50_ ≈ 50 µM) with increased expression of cytochrome c, cleavage of caspases-3 and −9. Given orally at a dose of 50 mg/kg/day (3 days a week) to mice xenografted with drug-resistant HCT-116 cell line, vitexin (**66**) reduced the growth of tumors (Bhardwaj et al. 2018).

Plants in the genus *Ficus* L. (1753) produce cytotoxic phenanthroindolizidine alkaloids such as ficuseptine (**69**) and tylophorine (**70**), from *Ficus septica* Burm.f., which at the concentration of 50 µM were toxic to gastric cancer cell line (NUGC) (Wu et al. 2002). 10*S*,13a*R*-Tylocrebrine *N*-oxide (**71**), 10*S*,13a*R*-isotylocrebrine *N*-oxide (**72**), and tylophorine (**70**), from *F. septica*, at the concentration of 10 µM, inhibited the proliferation of nasopharyngeal carcinoma cell line (HONE-1) by 92, 87, and 80%, respectively (Damu et al. 2009). Other cytotoxic principles in this genus are septicine-type alkaloids such as fistulopsines A (**73**) and B (**74**) from *Ficus fistulosa* Reinw. ex Blume which induced HCT-116 cell line cycle arrest in G1 phase (Yap et al. 2016).

### **Myrmecodia platytyrea** becc. (Rubiaceae)

To date, no cytotoxic principle appears to have been identified in this myrmecophytic epiphyte, although preliminary studies have demonstrated cytotoxic, anti-inflammatory, and immunomodulatory activities. Methanol extracts inhibited the growth of hepatoma cell line (Huh7) (Ju et al. 2018) and HepG2 cell line (IC_50_: 70 µg/mL), while being less toxic for Vero cell line (Zakaria and Aziz 2022). An extract was found to be cytotoxic to HepG2 cell line (IC_50_: 5.7 μg/mL) and induced apoptosis with cell cycle arrest in G_0_/G_1_ phase, increased expression of CDK2 and CDK5, Bax, and caspase-3. In mice, this extract given orally, was able to decrease the growth of hepatocellular carcinoma (Ibrahim 2021). The aqueous extract administered at a dose of 400 mg/kg induced anti-inflammatory effects and increased the immune system of mice (Mohd Zin, 2019). This activity could be due to polysaccharides and potentially β-glucans (Hasan et al. 2019). In line, Triana Hertiani and Sumardi (2010) observed the immunostimulant properties of an ethanol extract of domatia (or ant-tubers) of *Myrmecodia tuberosa* Jack and *Myrmecodia pendens* Merr. & L.M.Perry (collected in West Papua) (Ulfah et al. 2013). Plants in the genus *Myrmecodia* Jack (1823) produce iridoids (Hanh et al. 2016). The use of *M. platytyrea* by the Bajau for cancer treatment (Table 1) and the non-acute toxicity of its aqueous extract administered orally to rats (400 mg/kg/day) (Hasan et al. 2020) call for further experiments.

### **Paederia verticillata** Bl. (Rubiaceae)

Polar organic extracts of plants of the genus *Paederia* L. (1767) have demonstrated cytotoxic properties. This was observed with methanol extracts of leaves of *Paederia foetida* L. (MCF-7; IC_50_ : 550.1 µg/mL) (Morshed et al. 2012; Priyanto et al. 2022), and an ethanol extract of leaves of *Paederia lanuginosa* Wall. (HT-29; IC_50_: 28.8 µg/mL) (Hoang Phu et al. 2025).

**Figure 12.**
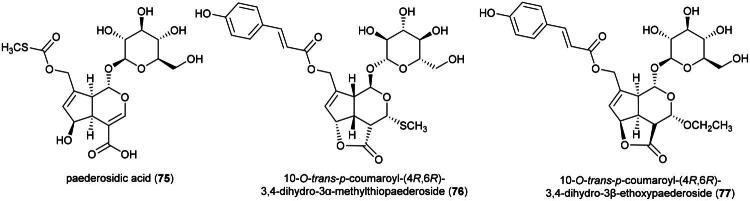
Cytotoxic iridoid glycosides of plants of the genus *Paederia.*

The cytotoxic principles isolated from these plants are mainly iridoids (Figure 12). For example, paederosidic acid (**75**) from *P. scandens* was found to be cytotoxic to gastric adenocarcinoma cell line (SGC-7901; IC_50_: 30.5 μM) and induced apoptosis with upregulation of caspases-3 and −9 as well as upregulation of Bax, and downregulation of Bcl-2 (Chen et al. 2015). We can also cite 10-*O*-*trans*-*p*-coumaroyl-(4*R*,6*R*)-3,4-dihydro-3*α*-methylthiopaederoside (**76**) toxic to five endocrine tumor cell lines (IC_50_ ˂ 20 µM) (Hu et al. 2024), as well as *trans*-*p*-coumaroyl-(4*S*,6*R*)-3,4-dihydro-3*β*-ethoxypaederoside (**77**) from the aerial parts of *Paederia yunnanensis* (H. Lév.) Rehder (Hu et al. 2025). The presence of cytotoxic iridoids in *P. verticillata* is probable and these remain to be identified.

### **Rennellia borneensis** baill. (Rubiaceae)

**Figure 13.**
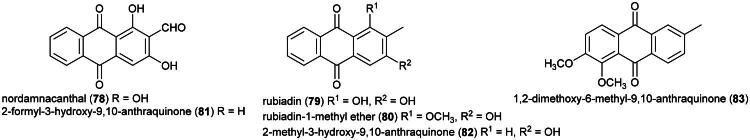
Cytotoxic anthraquinones of plants of the genus *Rennellia.*

Plants in the genus *Rennellia* Korth. (1851) produce cytotoxic anthraquinones (Figure 13). Examples are nordamnacanthal (**78**), rubiadin (**79**), and rubiadin-1-methyl ether (**80**) (Osman et al. 2010, 2016a). Nordamnacanthal (**79**) was found to be cytotoxic to oral squamous cell carcinoma cell line (H400 OSCC; IC_50_: 6.8 µg/mL) (Shaghayegh et al. 2017). Rubiadin (**79**) from *Morinda umbellata* L. inhibited the growth of HepG2 cell line (IC_50_: 3.6 µM) (Chiou et al. 2014) and from this plant rubiadin-1-methyl ether (**80**) inhibited the growth of MCF-7 cell line (IC_50_: 30 µg/mL) (Ali et al. 2000). Likewise, 2-formyl-3-hydroxy-9,10-anthraquinone (**81**), 2-methyl-3-hydroxy-9,10-anthraquinone (**82**), and 1,2-dimethoxy-6-methyl-9,10-anthraquinone (**83**) from the roots of *Rennellia elliptica* Korth. were active against MCF-7 cell line with IC_50_ values of 33, 50.1, and 38.7 µg/mL, respectively (Osman et al. 2016b). A preliminary study revealed the presence of anthraquinones in *R. borneensis* (Rushdan et al. 2023), endemic to Borneo, whose cytotoxic active principles remain to be identified. Since anthraquinones are mutagenic (Tikkanen et al. 1983), this shrub is unsuitable for any medicinal use.

### **Crassocephalum crepidioides** (benth.) S. Moore

**Figure 14.**
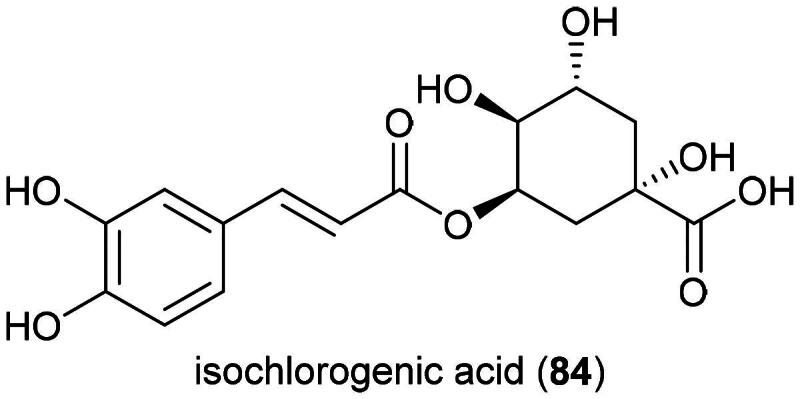
Cytotoxic natural products of plants of the genus *Kadsura.*

Aqueous extract of this herb (collected in Japan) given orally at a dose of 5 g/kg/day for 29 days to mice xenografted with S-180 cell line caused a decrease in tumor volume by about half. This extract did not cause cytotoxic effects *In vitro,* but when RAW264.7 cell line were treated with the extract at a concentration of 250 µg/mL, the culture supernatant could inhibit the growth of S-180 cell line because of the presence of nitric oxide derived from activation of NF-*κ*B and increased expression of iNOS. From this extract isochlorogenic acid (a caffeic acid derivative) (**84**) (Figure 14) activated NF-*κ*B in RAW264.7 cell line (Tomimori et al. 2012). Ethanol extract of the plant (collected in Malaysia) at the concentration of 25 µg/mL protected HepG2 against *tert*-butyl hydroperoxide (Wijaya et al. 2011).

*C. crepidioides* is used by the Murut against cancer and as a medicinal food by the Dusun (Table 1). The aqueous leaf extract administered orally gave an LD_50_ greater than 5 g/kg in rats (Nguemfo et al. 2021). However, the plant produces hepatotoxic pyrrolizidine alkaloids (Wachenheim et al. 1992).

### **Merremia gracilis** E.J.F. Campb. & argent (Convolvulaceae)

**Figure 15.**
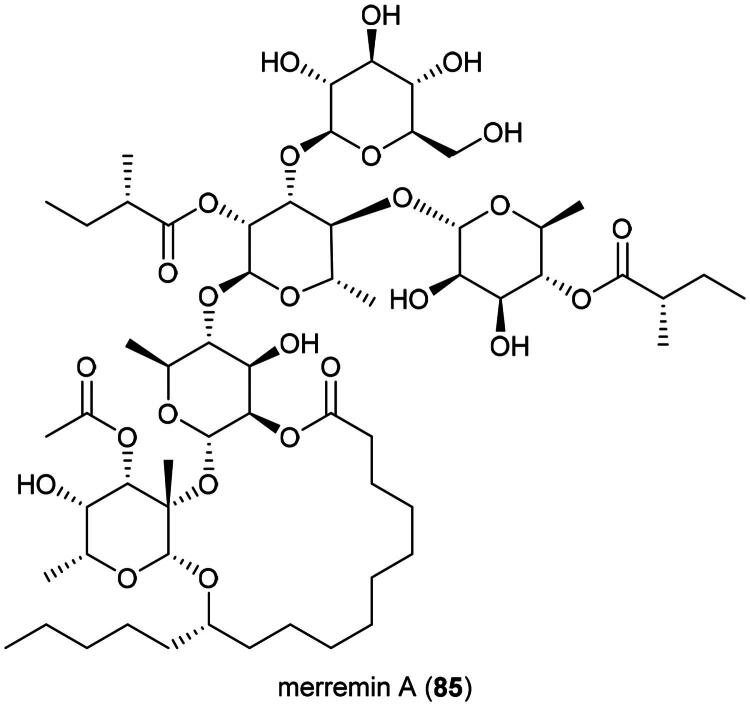
Cytotoxic caffeic acid derivative of *C. crepidioides.*

Plants of the genus *Merremia* Dennst. ex Endl. (1841) are known to be poisonous (Brito et al. 2019). They produce glycolipids some of which are able to increase the vulnerability of KB cell line to vinblastine such as merremin A (**85**) (Figure 15) from the aerial parts *Merremia hederacea* (Burm. f.) Hallier f. (Wang et al. 2014; Li et al. 2023). Ethanol extract of *Merremia emarginata* (Burm. f.) Hallier f. inhibited the growth of HT-29 cell line by 58% at the concentration of 200 µg/mL and induced apoptosis *via* increased expression of caspase-3, decreased expression of Bcl-2 and Bcl-xL (Benedict et al. 2024). Hexane extract of *M. emarginata* inhibited the growth of A549 cell line (IC_50_: 18.4 µg/mL) (Baskar et al. 2012).

### **Merremia peltata** (L.) merr. (Convolvulaceae)

The ethyl acetate extract of leaves was found to be cytotoxic to brine-shrimp s (LC_50_: 22 ppm) (Djamaan, 2016).

### Other plants used by multiple ethnic groups

*Airyantha borneensis* (Oliv.) Brummitt and *Koompassia malaccensis* Maing, have not been the subject of any phytochemical or pharmacological studies, as have all the plants of their respective genera.

## Plants used by the Lundayeh (Kelabit group)

The Lundayeh migrated from other parts of Borneo to Sabah about 100 years ago where they represent a minority. The Lundayeh are found in Sabah, and they are also known as Lunbawang in Sarawak and Kalimantan (King and King 1984). The wealth of their medicinal flora (especially the use of endemic plants) suggest that they should perhaps belong to the Bornean-speaking ethnic group close to the Murut (Wiart et al. 2025).

### **Kadsura borneensis** A.C. (Schisandraceae)

**Figure 16.**
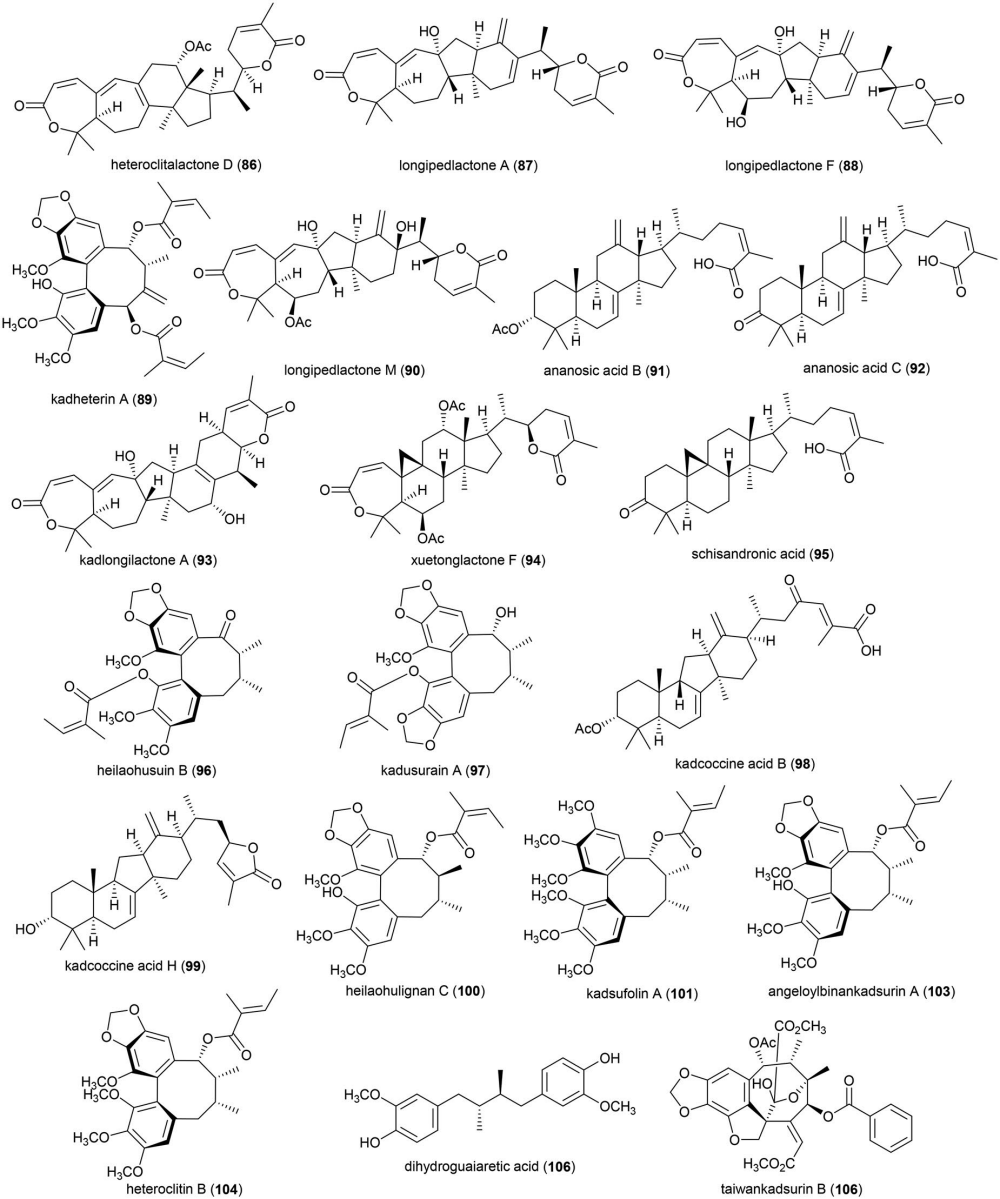
Cytotoxic glycolipid of plants in the genus *Merremia.*

Plants in the genus *Kadsura* Juss. (1810) produce a series of cytotoxic triterpenes rare in nature (Figure 16). Examples are heteroclitalactone D (**86**) (HL-60; IC_50_: 6.7 μM) (Wang et al. 2006), longipedlactone A (**87**) and F (**88**) (Xu et al. 2010), and kadheterin A (**89**) (HL-60; IC_50_: 14.5 μM) (Luo et al. 2017) from *Kadsura heteroclita* (Roxb.) Craib. Other examples are longipedlactone M (**90**) (Yang et al. 2010), ananosic acid B (**91**) (HeLa; IC_50_: 0.5 μg/mL), and ananosic acid C (**92**) (HeLa; IC_50_: 0.4 μg/mL) from *Kadsura ananosma* Kerr (Chen et al. 2004) (Figure 16). We can also cite kadlongilactone A (**93**) from *Kadsura longipedunculata* Finet & Gagnep. (Pu et al. 2007), xuetonglactone F (**94**) from *K. heteroclita* (BGC 823; IC_50_: 2 μM) (Shehla et al. 2020), and Schisandronic acid (**95**) from *Kadsura coccinea* (Lem.) A.C. Sm. Schisandronic acid (**95**) induced apoptosis in MCF-7 cell line (IC_50_: 8 μM) with activation of caspase-3 and cleavage of poly (ADP-ribose) polymerase (Tasneem et al. 2021).

Other cytotoxic principles in this genus are dibenzocyclooctadiene lignans, also rare in nature, such as heilaohusuin B (**96**) (Yang et al. 2019) and kadusurain A (**97**) from *Kadsura coccinea* (Lem.) A.C. Sm. (Zhao et al. 2014) as well as kadcoccine acid B (**98**) and H (**99**) (Hu et al. 2016). Heilaohulignan C (**100**) from *K. coccinea* induced apoptosis in gastric cancer cell line (BGC-823) and given to mice xenografted with gastric carcinoma cell line, caused a reduction of tumors (Daniyal et al. 2021). Additional instances of cytotoxic lignans are kadsufolin A (**101**), kadsufolin D (**102**), angeloylbinankadsurin A (**103**), and heteroclitin B (**104**) from *Kadsura oblongifolia* Merr. (Huang et al. 2011). In addition, a diarylbutane lignan *meso*-dihydroguaiaretic acid (**105**) was isolated from *K. heteroclita* (HT-29; IC_50_: 16.2 μM) (Minh et al. 2014) and taiwankadsurin B (**106**) (a homolignan) from *Kadsura philippinensis* Elmer (Shen et al. 2005). To date, nothing is known about the toxicity of *K. borneensis*, a vine endemic to Borneo.

### **Nepenthes ampullaria** jack (Nepenthaceae)

**Figure 17.**
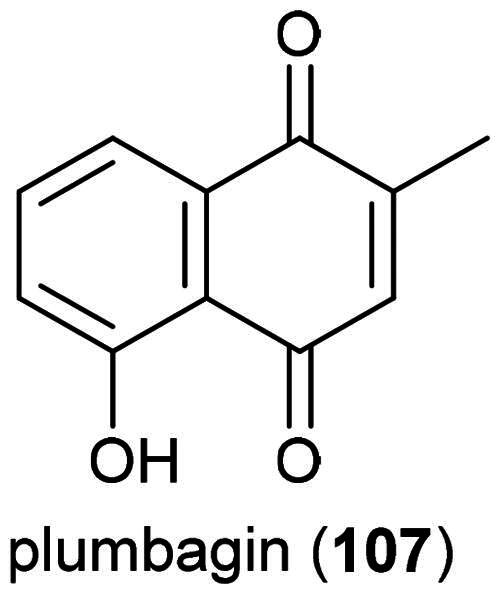
Cytotoxic naphthoquinone of plants of the genus *Nepenthes.*

An ethanol extract of the roots of this cultivable pitcher plant was found to be cytotoxic to HT-29 and Caco-2 cell line with IC_50_ values of 62.3 and 49.3 µg/mL, respectively, while being nontoxic to normal intestinal cell line (CCD841CoN) (IC_50_ > 512 µg/mL) (Dřímalová, 2024). Cytotoxic principles have not been isolated from this plant. What we know about plants of the genus *Nepenthes* L. (1753) is that their polar organic extracts are often cytotoxic owing to the presence of naphthoquinones. A methanol extract of stems of a plant in the genus (purchased in Taiwan) was found to be cytotoxic to gingival carcinoma cell line (Ca9-22) (IC_50_ ≈ 15 µg/mL) and at the concentration of 80 µg/mL induced apoptosis with cell cycle arrest in G_2_ phase (Lin et al. 2023). Plumbagin (**107**) (Figure 17), a naphthoquinone isolated from the roots of *Nepenthes alata* Blanco (purchased in Korea), induced cell cycle arrest in the G_2_/M phase of MCF-7 cell line, as well as apoptosis *via* increased production of reactive oxygen species, elevation in the ratio of Bax/Bcl-2, and release of cytochrome c. In mice xenografted with MCF-7 cell line, plumbagin (**107**) caused a decrease in the growth of tumors (De et al. 2019). *N. ampullaria* probably produces cytotoxic naphthoquinones. These remain to be identified. Naphthoquinones, like plumbagin (**107**), are poisonous (Teixeira et al. 2024), which renders *N. ampullaria* unfit for any nutraceutical development.

### **Symplocos odoratissima** choisy ex zoll. (Symplocaceae)

**Figure 18.**
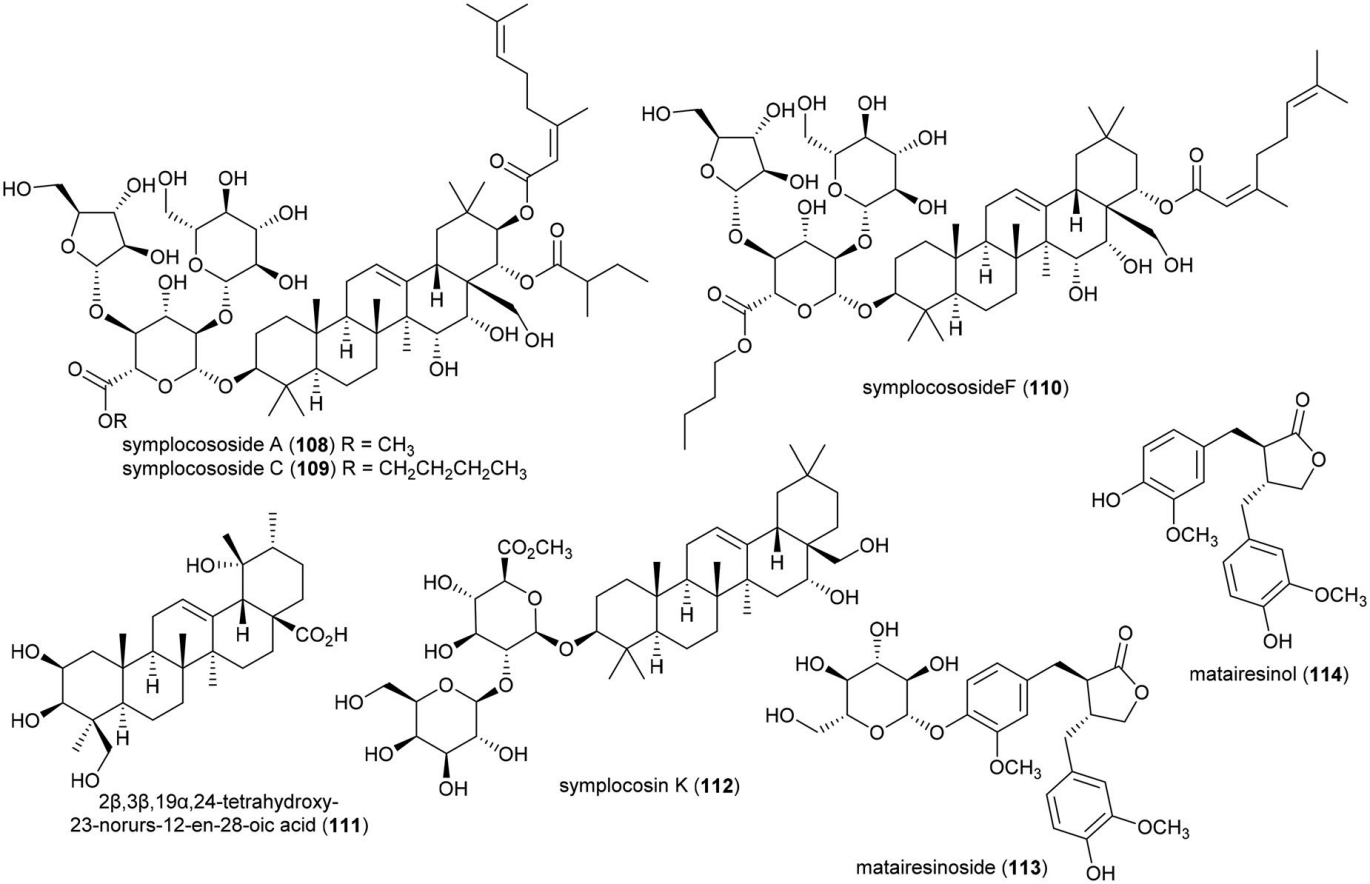
Cytotoxic natural products of plants of the genus *Symplocos.*

Cytotoxic triterpene saponins have been identified from *Symplocos chinensis* (Lour.) Druce (Fu et al. 2006), including symplocososides A (**108**), C (**109**), and F (**110**) (Tang et al. 2004) and as well as 2β,3β,19-α,24-tetrahydroxy-23-norurs-12-en-28-oic acid (**111**) (an ursane-type triterpene), the latter active against BGC-823 cell line (Li et al. 2003) (Figure 18). From the leaves of *Symplocos cochinchinensis* (Lour.) S.Moore (collected in Japan), symplocosin K (**112**) was found to be cytotoxic to A549 cell line (IC_50_: 73.8 μM) (Ohyama et al. 2020).

Phenolic glycosides have been identified from *Symplocos racemosa* Wight ex C.B.Clarke (collected in Pakistan) (Choudhary et al. 2004) and may account for the fact that a butanol extract of the bark of this plant inhibited the growth of HL-60 and HeLa cell line with IC_50_ values of 27.1 and 22.8 μg/mL, respectively (Raval et al. 2009). Other constituents in this genus are *seco*-iridoids from *S. cochinchinensis* (Lee et al. 2019), dibenzylbutyrolactone lignan glycosides such as matairesinoside (**113**) from the roots of *Symplocos caudata* Wall. ex G.Don (collected in China) (Huo et al. 2008) or dibenzylbutyrolactone lignans as in matairesinol (**114**) (CaCo-2; IC_50_: 220 μM) (Shoeb et al. 2007) from *Symplocos setchuensis* Brand as well as, from this plant, pyridoindole alkaloids (Ishida et al. 2001). *S. racemosa* produces anthraquinones (Farooq et al. 2017). Matairesinol (**114**) was found to be cytotoxic to HepG2 cell line (IC_50:_ 30.8 µM) and induced apoptosis with mitochondrial dysfunction, an increase in reactive oxygen species, and activation of caspase-3 (Arzuk et al. 2024). *S. odoratissima* has not been studied phytochemically or pharmacologically and could be toxic since plants of the genus *Symplocos* Jack (1760) are aluminum accumulators (Schmitt et al. 2016).

### **Hydnophytum formicarum** jack (Rubiaceae)

**Figure 19.**
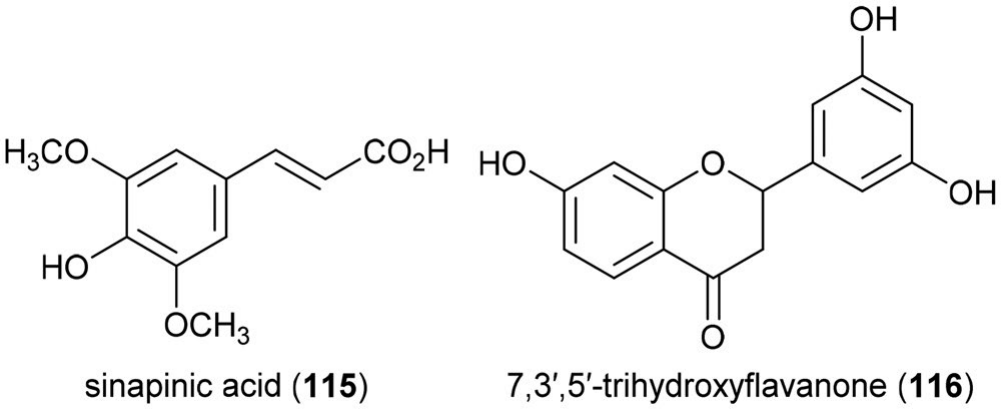
Cytotoxic phenolic compounds of *H. formicarium.*

The data available so far indicates the presence of cytotoxic phenolics in this myrmecophyte epiphyte, but these have mild effects *in vitro*, which suggests that use as a treatment for cancer could be due, at least in part, to the presence of immunostimulant principles. Polar organic extract of domatia inhibited the growth fibrosarcoma cell line (HT-1080; IC_50_: 9.9 µg/mL) (Ueda et al. 2002) and the activity of histone deacetylase *in vitro* (4 µg/mL; 40%). The plant produces sinapinic acid (**115**) (a phenylpropanoid) (Figure 19), which inhibited histone deacetylase activity with an IC_50_ value of 2.2 mM and inhibited the survival of HeLa cell line (Senawong et al. 2013). Another cytotoxic phenolic identified from *H. formicarium* is 7,3′,5′-trihydroxyflavanone (**116**), which caused DNA fragmentation and apoptosis in MCF-7 cell line *via* increased expression of Bax, decreased expression of Bcl-2 at the concentration of 15 μg/mL (Abdullah et al. 2010).

Of note, an ethanol extract of domatia of *H. formicarum* (collected in West Papua) inhibited the growth of T-47D cell line by 53.4% (100 µg/mL) and induced lymphocyte proliferation (Darwis et al. 2014). An aqueous extract of domatia given orally to mice caused some immunomodulatory properties (Putra et al. 2025). Do they produce β-glucans?

*H. formicarum* is cultivable (Table 2). The immunostimulatory properties of this epiphytic plant could be due to the presence of β-glucans or other polysaccharides. More experiments are needed.

### **Jasminum bifarium** wall (Oleaceae)

**Figure 20.**
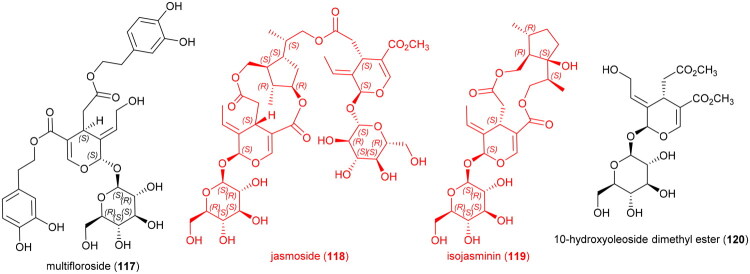
Cytotoxic iridoid glycosides of *J. bifarium.*

Plants in the genus *Jasminum* L. (1753) produce cytotoxic seco-iridoids. We can cite multifloroside (**117**) (Figure 20) from *Jasminum multiflorum* (Burm. f.) Andrews decreased the viability of epidermoid carcinoma cell line (A431) (≈ 80%) at the concentration of 200 µM, cell cycle arrest in S phase, an increase of reactive oxygen species, and induction of apoptosis (Zhang et al. 2019). From *Jasminum humile* L., jasmoside (**118**) and isojasminin (**119**) were found to be cytotoxic to THP-1 cell line with IC_50_ values of 27.5 and 51 µg/mL, respectively (Mansour et al. 2022). 10-Hydroxyoleoside dimethyl ester (**120**) from *Jasminum lanceolarium* Roxb. (Shen et al. 1997) was found to be marginally cytotoxic to A431 cell line (200 µM) (Zhang et al. 2019).

**Figure 21.**
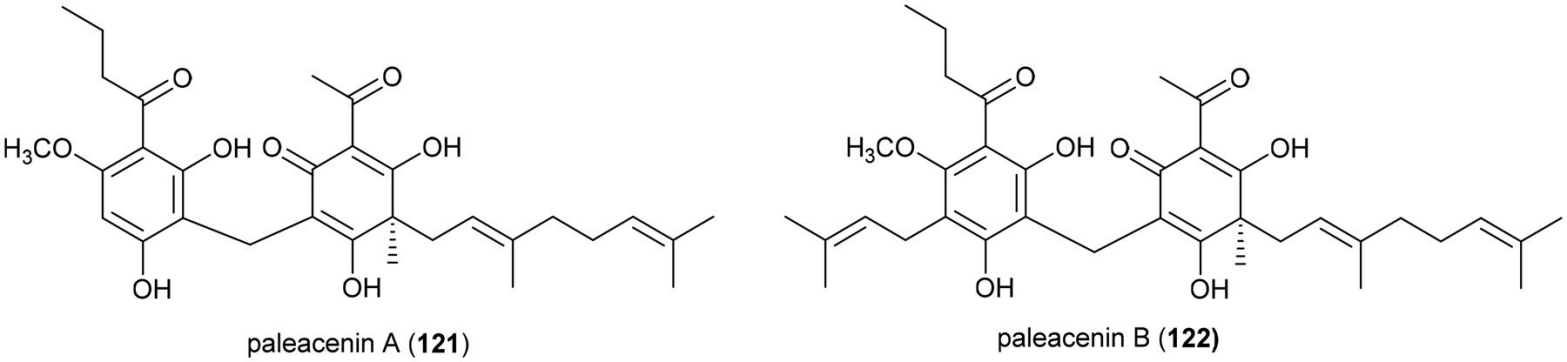
Cytotoxic prenylated prenylated phloroglucinols from plants of the family polypodiaceae.

Another interesting aspect of these plants is that their polar and med-polar organic extracts are capable of preventing the development of tumors in rodents resulting from exposure to 7,12-dimethylbenz[*a*]anthracene. This was observed with an ethanolic extract of flowers of *Jasminum grandiflorum* L. (300 mg/kg for 14 weeks) (Kolanjiappan and Manoharan 2005), an ethyl acetate extract of leaves of *Jasminum subtriplinerve* Blume (14.4 mg/kg for four weeks) (Minh et al. 2024), and the essential oil of *Jasminum sambac* (L.) Aiton (10 mL/kg; orally for 45 days) (Lakshmi et al. 2024).

### Other plants used by the Lundayeh

*Lygodium salicifolium* C. Presl. (Lygodiaceae) and *Garnotia acutigluma* (Steud.) Ohwi have not been the subject of any phytochemical or pharmacological studies.

## Plants used by the Brunei (Malayic group)

### **Drymoglossum piloselloides** (L.) C. Presl (Polypodiaceae)

An ethanol extract inhibited the growth of MCF-7 cell line (IC_50_: 83.6 µg/ml) (Endrini 2009), HeLa cell line (IC_50_: 16.2 µg/ml) (Su’lain et al. 2019), and P388 cell line (IC_50_: 19.3 µg/ml) (Sahid et al. 2013). The plant produces a series of prenylated phloroglucinols (Socolsky et al. 2010, 2011), which could be examined for their possible cytotoxic effects since the acylphloroglucinols paleacenins A (**121**) and B (**122**) from *Elaphoglossum paleaceum* (Hook. & Grev.) Sledge (Polypodiaceae) were cytotoxic to PC-3 cell line with IC_50_ values of 1.7 and 2.9 µM, respectively (Arvizu-Espinosa et al. 2019) (Figure 21).

### **Gnetum macrostachyum** hook.f. (Gnetaceae)

**Figure 22.**
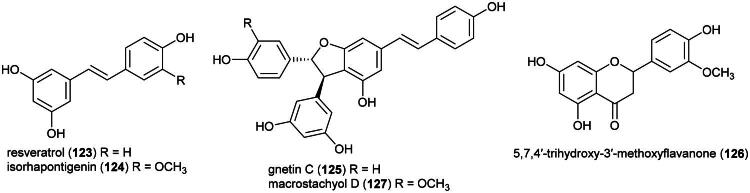
Cytotoxic phenolic compounds from *G. macrostachyum.*

This stout primary forest climber is employed for fatigue (Table 1) which is one of the symptoms of cancer. It produces the stilbenes resveratrol (**123**) and isorhapontigenin (**124**), and the stilbenoid gnetin C (**125**) (Kloypan et al. 2012), 5,7,4′-trihydroxy-3′-methoxyflavanone (**126**) (Saisin et al. 2009) (Figure 22). Resveratrol (**123**) and isorhapontigenin (**124**) inhibited the growth of MCF-7 and T-47D cell line with the IC_50_ values of 51.1 μM (Alkharashi, 2023) and 40 μM (Subedi et al. 2019), respectively. Gnetin C (**125**) inhibited the growth of prostate cancer (DU-145) cell line (IC_50_: 6.6 μM). Given intraperitoneally at a dose of 50 mg/kg/day for 30 days to mice xenografted with prostate cancer cell line (C3M-Luc), gnetin C (**125**) caused a decrease in tumor growth (Gadkari et al. 2020). A very interesting point is that gnetin C (125), when mixed into the diet of rats with prostate cancer (70 mg/kg) for 17 weeks, induced some levels of protection (Parupathi et al. 2022). If this activity is confirmed by further studies and if this stilbenoid proves nontoxic, there would be grounds for clinical development. The stilbenoid macrostachyol D (**127**) from the roots was found to be cytotoxic to cervical cancer cell line (HeLa) (IC_50_: 4.1 μM) (Sri-in et al. 2011).

## Plants used by the Bajau (Bajau group)

The Bajau are categorized as west coast Bajau (Bajau Samah) or the east coast Bajau and in available data pertaining to the medicinal plant they use the separation does not exist. They tend to use plants growing in mangroves and sea side. These plants are in general well-known and non-endemic (Wiart et al. 2025).

### **Diplazium cordifolium** Bl. (Athyriaceae)

This fern does not appear to have been phytochemically or pharmacologically studied. Toxicological studies on this plant have not yet to be conducted.

### **Dischidia rafflesiana** wall. (Asclepiadaceae)

**Figure 23.**
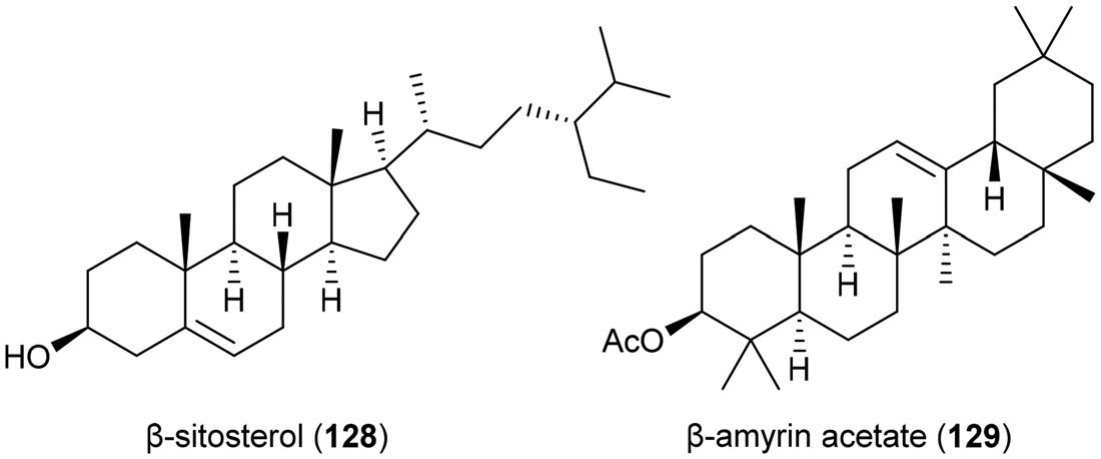
Cytotoxic steroid and triterpene from plants of the genus *Dischidia.*

This cultivable climber affords a remedy for cancer (Table 1 and 2). It is known to produce β-amyrin (**63**) (Van Hoang et al. 2022). Methanol extract of a plant in the genus *Dischidia* R. Br. (1810) was found to be cytotoxic to P388 cell line (IC_50_: 24.8 µg/mL) (Manggribeth et al. 2019). Hexane and dichloromethane extracts of *Dischidia nummularia* R.Br. were found to be cytotoxic to MDA-MB-231 cell line (Khalil-ur-Rehman et al. 2019). From this plant, β-sitosterol (**128**) (Figure 23) was found to be cytotoxic to P-388 cell line (IC_50_: 0.5 µM) (Benu et al. 2023). From *Dischidia alboflava* Costantin (collected in Vietnam) cytotoxic triterpenes have been identified such as β-amyrin acetate (**129**), friedelin (**53**), and lupeol (**54**) (Linh et al. 2023). β-Amyrin acetate (**129**) (Figure 23) inhibited the growth of A2780 cell line (Chaturvedula et al. 2002).

## Plants used by the Dusun (Bornean group, Dusunic family)

### General observations

Regarding the medicinal plant species used by the Dusun with chemotherapeutic potential, the following observations can be made: (i) half of them are used as edible plants, (ii) they use four species of ferns, (iii) these plants are often from primary forest, and (iv) six species are endemic.

### **Gleichenia truncata** (Willd.) Spreng. (Gleicheniaceae)

**Figure 24.**
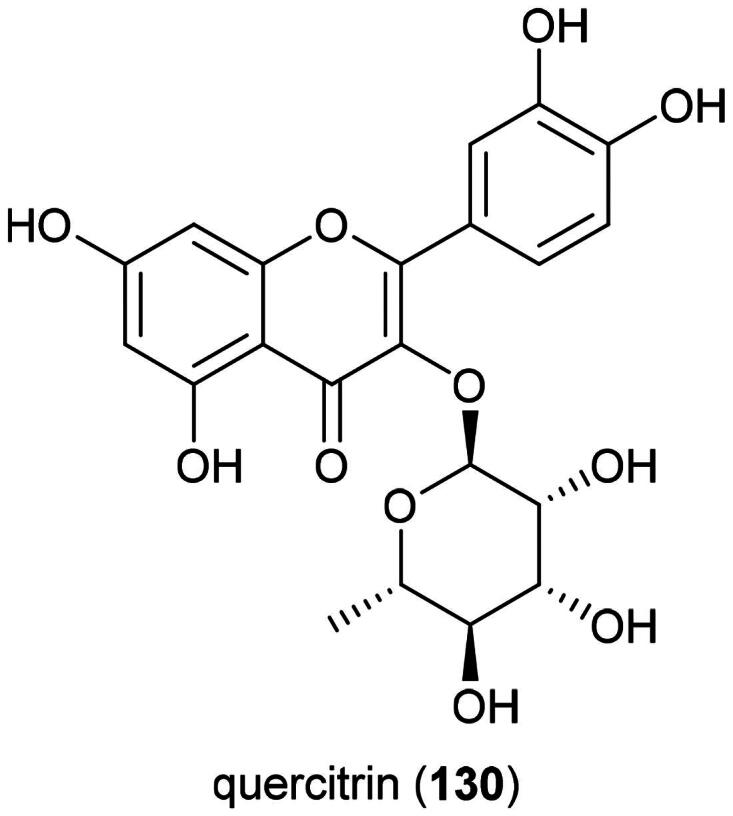
Cytotoxic flavonol glycoside from plants of the family Gleicheniaceae.

Labdane glycosides are produced by *Gleichenia quadripartita* (Poir.) T. Moore (Socolsky et al. 2007) and *Gleichenia japonica* Spreng.(Munesada et al. 1992) and clerodane glycosides by *Gleichenia microphylla* R.Br. (Wada et al. 1998). *Gleichinia alpinia* R. Br. (collected in Australia) produces flavonol glycosides such as rutin (**21**) (Gyeltshen et al. 2022) In the family Gleicheniaceae, *Dicranopteris linearis* (Burm. f.) Underw. produces quercitrin (**130**) (Figure 24) toxic to HL-60 cell line (IC_50_: 4.5 µg/mL) (Chen et al. 2014).

### **Diplazium esculentum** (retz.) Sw

**Figure 25.**
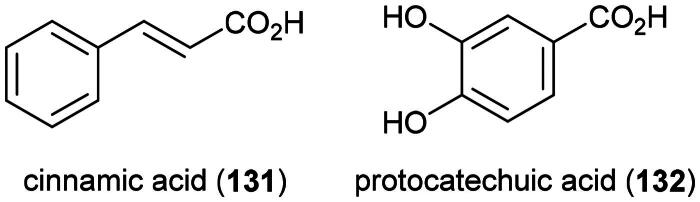
Cytotoxic phenolic compounds of *D. esculentum.*

Organic polar extracts of this fern demonstrated cytotoxic effect against MDA-MB-231 cell line (ethanol, 100 µg/mL) (Rahmat et al. 2003), brine-shrimp (methanol, LC_50_: 1.6 µg/mL) (Akter et al. 2014), and chronic myelogenous leukemia cell line (K562) (methanol, 500 µg/mL) (Salleh and Ab Latif 2022). The plant produces simple phenolics (Gyeltshen et al. 2022), cinnamic acid (**131**) (phenyl propanoid), protocatechuic acid (132) (a benzoic acid derivative), and rutin (**21**), as well as ecdysteroids (Watanabe et al. 2021) (Figure 25). Cinnamic acid (**131**) is an inhibitor of histone deacetylase (IC_50_: 9.1 µg/mL) (Koyu et al. 2024) and abrogated the survival of A549 cell line. Given orally three times a week for a total of six doses at a dose of 1.5 mmol/kg to mice xenografted with HT-29 cell line, cinnamic acid (**131**) evoked a decrease in the volume of tumors by about half (Zhu et al. 2016). Protocatechuic acid (132) prevented the development of mutations induced by H_2_O_2_ in common fruit flies (Anter et al. 2011).

Although this fern is a medicinal food (Table 1) (Maid et al. 2017), caution should be exercised regarding the nutraceutical development of this plant. Preliminary data suggest toxic effects greater than 2.5 g/kg (chloroform extract) in mice (Salleh and Ab Latif 2022), but oral administration of an aqueous extract given to mice for 180 days caused erythrocyte destruction and immunosuppression (Roy et al. 2013).

### **Nephrolepis acutifolia** (Desv.) Christ (Nephrolepidaceae)

Aqueous extract of this epiphyte fern was found to be cytotoxic to K562 cell line (IC_50_: 190.8 µg/mL) (Chai et al. 2015), and the cytotoxic principles involved have not been identified. Essential oil from the roots of *Nephrolepis exaltata* (L.) Schott and *Nephrolepis cordifolia* (L.) C. Presl inhibited the growth of A549 cell line with IC_50_ values of 24.3 and 23.6 µg/mL, respectively (El-Tantawy et al. 2015), while an acetone extract was found to inhibit the growth of PC-3 cell line (Bobach et al. 2014). *N. acutifolia* is used as medicinal food (Table 1). More experiments are needed.

### **Acrostichum aureum** L. (Pteridaceae)

**Figure 26.**
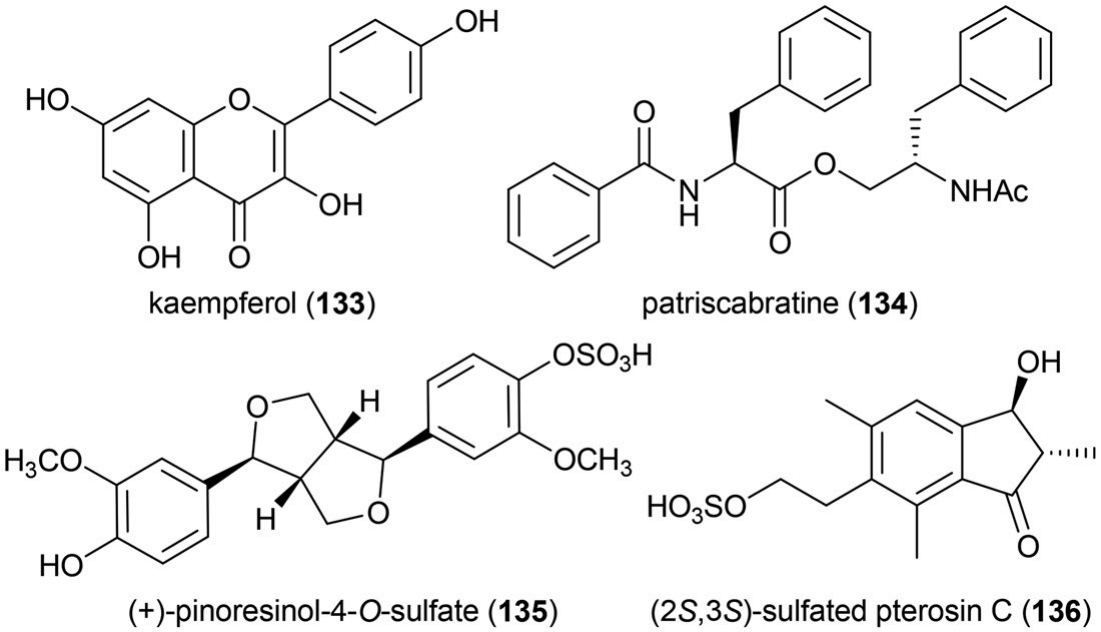
Cytotoxic compounds of *A. aureum.*

An ethyl acetate extract was found to be cytotoxic to HeLa cell line (Dai et al. 2005). This fern produces rutin (**21**), kaempferol (**133**), and an *N*-benzoylphenylalanine derivative patriscabratine (**134**) (Figure 26). Patriscabratine (**134**) was found to be cytotoxic to MDA-MB-231 and MCF-7 cell line, with IC_50_ values of 69.8 and 197.3 μM, respectively (Uddin et al. 2012). Kaempferol (**133**) inhibited the growth of ovarian cancer cell line (A2780; IC_50_: 19 μM) (Pham et al. 2018).

Other cytotoxic principles include the lignan (+)-pinoresinol-4-*O*-sulfate (**135**) (MCF-7; IC_50_: 7 µM) (Minh et al. 2022) and (2*S*,3*S*)-sulfated pterosin C (**136**) (a norsesquiterpene) (AGS; IC_50_: 23.9 μM) (Figure 26) (Uddin et al. 2011). The plant has anti-inflammatory properties and this is interesting because chronic inflammation is one of the etiological factors of cancer. An ethanol extract given to mice orally at a dose of 400 mg/kg to assuage the pain caused by injection of acetic acid as effectively as diclofenac (25 mg/kg) (Khan et al. 2013). An aqueous extract of the aerial part given orally to rats at a dose of 400 mg/kg/day for 7 days, prior to oral administration of absolute ethanol, prevented the formation of gastric ulcers (Wu et al. 2018).

*A. aureum* is taken as medicinal food (Table 1). Preliminary toxicological studies indicate a lack of toxicity in acute (LD_50_ > 5 g/kg) and sub-acute (750 mg/kg/day for 28 days) studies (Akinwumi et al. 2024). It should be noted, however, that an ethanolic extract administered to rats on days 1 to 7 after coitus prevented all pregnancies (Dhar et al. 1992).

### **Goniothalamus roseus** Stapf

**Figure 27.**
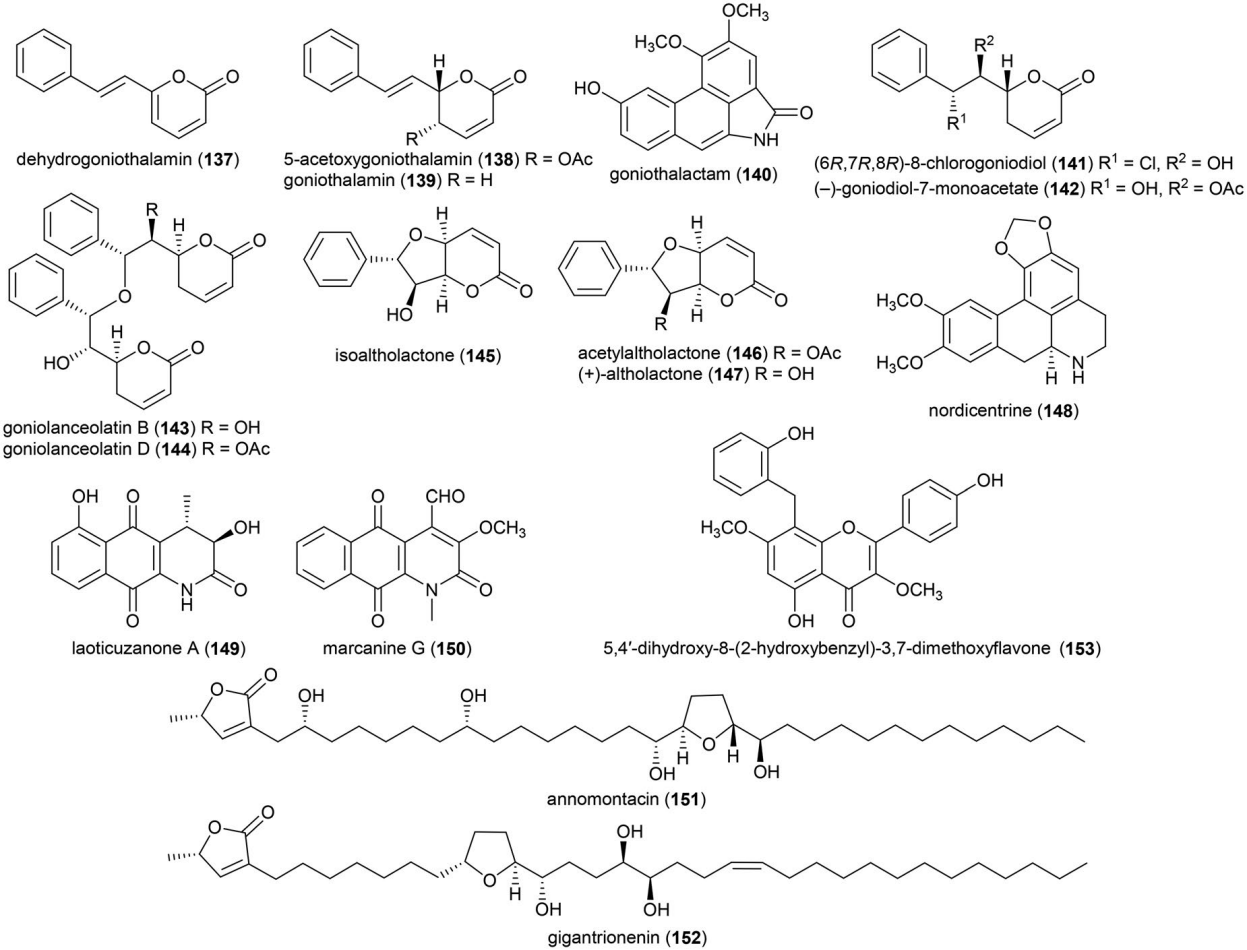
Cytotoxic compounds of plants in the genus *Goniothalamus.*

This endemic rainforest treelet produces nephrotoxic aristolactam alkaloids (Xue et al. 2025) and has therefore no potential for the development of a herbal remedy. Rather, it is a source of cytotoxic principles such as the styrylpyrones dehydrogoniothalamin (**137**) (PC-3; IC_50_: 90.4 µM) (de Souza et al. 2021), 5-acetoxygoniothalamin (**138**) (HCT-116; IC_50_: 8.6 μM) (Meesakul et al. 2020), goniothalamin (**139**) (HL-60; IC_50_: 5.6 µM) (Petsophonsakul et al. 2013), and goniothalactam (**140**) (an aristolactam alkaloid) (P-388; IC_50_ < 4 μg/mL) (Tsai et al. 2005) (Figure 27).

Cytotoxic styrylpyrones are rare in nature and mostly found in the genus *Goniothalamus* (Blume) Hook. f. & Thomson (1855). We can cite (6 *R*,7*R*,8*R*)-8-chlorogoniodiol (**141**) from *Goniothalamus amuyon* (Blanco) Merr. (Lan et al. 2003), (–)-goniodiol-7-monoacetate (**142**) from *Goniothalamus repevensis* Pierre ex Finet & Gagnep. (ASK; IC_50_: 10.2 µM) (Chanakul et al. 2022), and goniolanceolatin B (**143**) and D (**144**) (styrylpyrone dimers) from *Goniothalamus lanceolatus* Miq. (Bihud et al. 2019).

Other cytotoxic principles are furanopyrones such as isoaltholactone (**145**) and aporphine alkaloid such as liriodenine (**37**) from *Goniothalamus gitingensis* Elmer (collected in the Philippines), toxic to K562 cell line with IC_50_ values of 4.3 and 6.1 µg/mL, respectively (Macabeo et al. 2014). From *Goniothalamus laoticus* (Finet & Gagnep.) Bân (collected in Thailand) acetylaltholactone (**146**), (+)-altholactone (**147**), and nordicentrine (**148**) (an aporphine alkaloid) were toxic to KB cell line with IC_50_ values of 2.9, 3.5, and 0.4 µg/mL, respectively (Lekphrom, et al. 2009). From this plant, the azaanthraquinone alkaloid laoticuzanone A (**149**) inhibited the growth of KB and HeLa cell lines with IC_50_ values of 0.6 and 0.5 μg/mL, respectively (Tip-Pyang et al. 2010). From *Goniothalamus marcanii* Craib, another azaanthraquinone, marcanine G (**150**), was found to be cytotoxic to A549 and MCF-7 cell line, with IC_50_ values of 14.8 and 15.1 μM, respectively (Thanuphol et al. 2018). Other cytotoxic principles in the genus are acetogenins such as annomontacin (**151**) and gigantrionenin (**152**) from Goniothalamus giganteus Hook. f. & Thomson (Fang et al. 1992), as well as flavonols such as 5,4′-dihydroxy-8-(2-hydroxybenzyl)-3,7-dimethoxyflavone (**153**) (HepG2; IC_50_: 16.7 μM) (Trieu et al. 2021).

### **Goniothalamus velutinus** Airy Shaw

**Figure 28.**
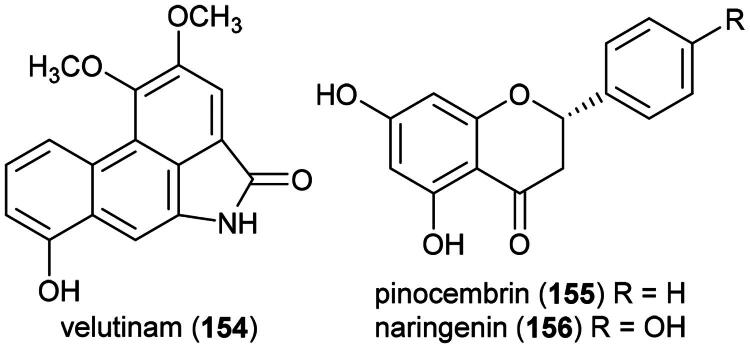
Cytotoxic compounds of *G. velutinus.*

This endemic primary rainforest produces nephrotoxic aristolactam alkaloids (aristolactam I, aristolactam AII, and aristolactam BII) (Iqbal et al. 2018). A methanol extract of bark (collected in Brunei) at a concentration of 50 μg/mL abrogated the survival of brine-shrimp s and inhibited the survival of A549 cell line with an IC_50_ value of 26.3 μg/mL, and induced apoptosis (Erum et al. 2016). From the stems (collected in Brunei), velutinam (**154**) (Figure 28) inhibited the proliferation of cervical carcinoma cell line (CaSki) with an IC_50_ value of 10.9 μg/mL (Iqbal et al. 2018), but is nephrotoxic (Xue et al. 2025). Other compounds found in the bark (collected in Sarawak) were the flavanones, pinocembrine (**155**) and naringenin (**156**), as well as goniothalamin (**139**) (Ahmad et al. 2006). Pinocembrine (**155**) was found to be cytotoxic to drug-resistant acute lymphoblastic leukemia cell line (CEM/ADR5000) (IC_50_: 53.5 μM) (Joray et al. 2015). In mice xenografted with mouse fibrosarcoma cell line (S-180), the daily intraperitoneal administration of naringenin (**156**) at a dose of 300 mg/kg for five days caused a decrease in tumor weight by about 50% (Kanno et al. 2005).

### **Polyalthia tenuipes** Merr. (Annonaceae)

**Figure 29.**
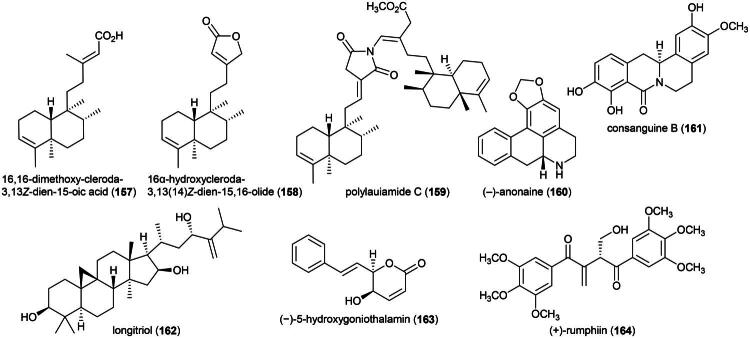
Cytotoxic natural products from plants of the genus *Polyalthia.*

Plants of the genus Polyalthia Blume (1930) are a prolific source of diterpenes and cytotoxic isoquinoline alkaloids. As for the diterpenes we can cite 16,16-dimethoxy-cleroda-3,13-*Z*-dien-15-oic acid (**157**) (Figure 29) from the leaves of *Polyalthia simiarum* (Buch.-Ham. ex Hook. f. & Thomson) Benth. ex Hook. f. & Thomson (collected in China) (SMMC-7721; IC_50_: 22.4 μM) (Duan et al. 2020), 16*α*-hydroxycleroda-3,13(14)*Z*-dien-15,16-olide (**158**) from *Polyalthia peteloti* Merr. (Yang et al. 2016) and *Polyalthia barnesii* Merr. (Ma et al. 1994), and polylauiamide C (**159**) (a clerodane diterpene dimer derivative) isolated from the roots of *Polyalthia laui* Merr. (HeLa: IC_50_: 25.1 μM) (Yu et al. 2016).

An aporphine alkaloid, (–)-anonaine (**160**), from the leaves of *Polyalthia longifolia* var. *pendula* Benthall (collected in Taiwan), abrogated the survival of AGS cell line (IC_50_: 8.6 µM) (Chen et al. 2000). From the stems and leaves of *Polyalthia obliqua* Hook.f. & Thomson (collected in China), consanguine B (**161**) (an oxoprotoberberine alkaloid) was found to be cytotoxic to HeLa and MCF-7 cell lines with IC_50_ values of 24.1 and 33.5 μM, respectively (Wu et al. 2016).

Longitriol (**162**), a cycloartane-type triterpene from the leaves of *P. longifolia* var. *pendula*, inhibited the growth of uterine cancer cell line (C33A) and A549 cell line with IC_50_ values of 10 and 13.1 µg/mL, respectively (Sashidhara et al. 2010). Other compounds are (−)-5-hydroxygoniothalamin (**163**) (a styrylpyrone) from the leaves of *Polyalthia parviflora* (collected in Vietnam) (A549, IC_50_: 7.9 μM) (Liou et al. 2014) and (+)-rumphiin (**164**) (a phenylpropanoid dimer) isolated from the stems of *Polyalthia rumphii* (Blume ex Hensch.) Merr. (collected in China) (K562, IC_50_: 63.2 µg/mL) (Wang et al. 2013).

Organic polar and mid-polar extracts of plants in this genus have been reported to be cytotoxic from *Polyalthia cerasoides* (Roxb.) Bedd. (ethanol; L929; IC_50_ ≈ 40 µg/mL) (Ravikumar et al. 2008), *Polyalthia debilis* (Pierre) Finet & Gagnep. (chloroform extract; HepG2; 23 µg/mL) (Prachayasittikul et al. 2009), *Polyalthia evecta* (Pierre) Finet & Gagnep. (ethanol; HepG2; IC_50_: 62.8 µg/mL) (Macana et al. 2012). Essential oil of stems of *Polyalthia suberosa* (Roxb.) Thwaites was found to be cytotoxic to MCF-7 cell line (IC_50_: 66.7 μg/mL) (The et al. 2021).

### **Eriocaulon longifolium** Nees ex Kunth (Eriocaulaceae)

**Figure 30.**
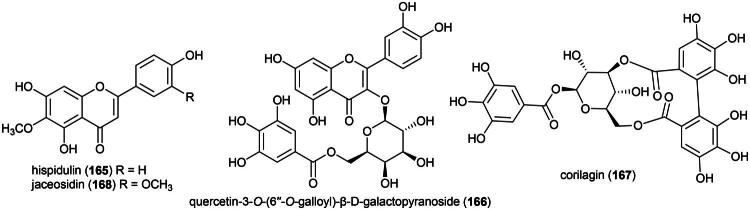
Cytotoxic phenolic compounds from plants of the genus *Polyalthia.*

Ethyl acetate extract of the whole plant (purchased in China) inhibited the growth of K562 cell line (IC_50_ ≈ 40 µg/L), and induced apoptosis with cell cycle arrest in G_0_/G_1_, inhibition of aurora kinases A and B kinase, upregulation of p53 and Bax, downregulation of Bcl-2, activation of caspases-3 and −9, release of cytochrome c, and cleavage of poly (ADP-ribose) polymerase (Fan et al. 2016). From this plant, hispidulin (**165**) (a flavone), quercetin-3-*O*-(6″-*O*-galloyl)-β-D-galactopyranoside (**166**) (a flavone glycoside), and corilagin (**167**) (an ellagitannin) inhibited the proliferation of HepG2 cell line with IC_50_ values of 17.8, 11.2, and 16.6 μM, respectively and induced apoptosis (Fan et al. 2015) (Figure 30).

A dichloromethane extract of *Eriocaulon cinereum* R.Br. (collected in Indonesia) was found to be moderately cytotoxic to MCF-7 cell line (IC_50_: 101.1 µg/mL) (Nugraha et al. 2021). From the capitula of *Eriocaulon australe* R.Br. (collected in China), hispidulin (**165**) and jaceosidin (**168**) (a flavone) were found to be cytotoxic to MCF-7 cell line with IC_50_ values of 7.6 and 15.6 µg/mL, respectively (Xu et al. 2013).

### **Dendrocalamus asper** (Schult. f.) Backer ex K. Heyne (Poaceae)

An ethanolic extract of edible young shoots (harvested in Sulawesi) inhibited the growth of MCF-7 cell line (IC_50_: 1.4 µg/mL) (Ontaha et al. 2021). The active compounds responsible for this activity are not yet known. However, it is known that plants of the genus *Dendrocalamus* Nees (1835) produce rutin (**21**) (Luo et al. 2022). The plant, which is cultivable (Table 2), produces cyanogenic compounds (Pattarathitiwat et al. 2021); the Dusun mitigate its toxicity by boiling it prior to consumption.

### **Panicum palmifolium** J. Koenig (Poaceae)

**Figure 31.**
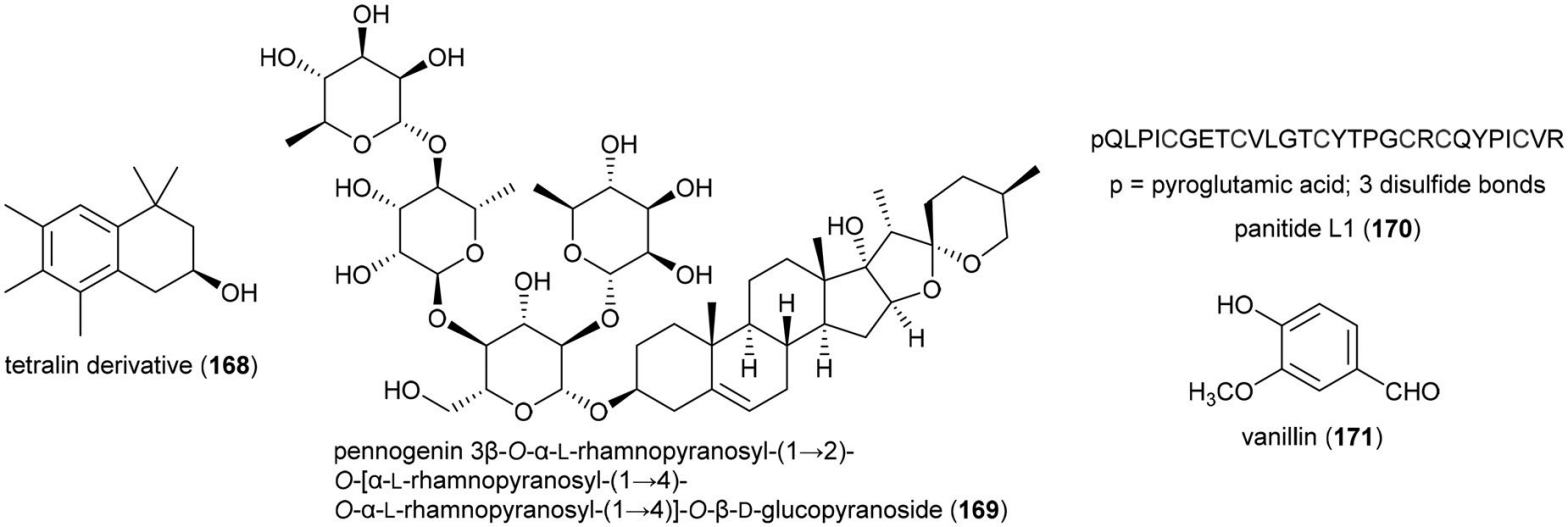
Cytotoxic compounds from plants of the genus *Panicum.*

An extract of the plant (collected in the Philippines) showed some activity against KB cell line (Spjut, 2005). The molecules responsible for this activity are unknown, but phenolic compounds, saponins, and cyclotides can be expected (Figure 31). A tetralin derivative (**168**) isolated from *Panicum turgidum* Forssk. was found to be cytotoxic against ovarian carcinoma (SK-OV-3) and breast carcinoma cell line (BT-549) with IC_50_ values of 5.6 and 10.3 µg/mL, respectively (Zaki et al. 2023). *P. turgidum* also produces cytotoxic steroidal saponins such as pennogenin 3β-*O*-α-L-rhamnopyranosyl-(1→2)-*O*-[α-L-rhamnopyranosyl-(1→4)-*O*-α-L-rhamnopyranosyl-(1→4)]-*O*-β-D-glucopyranoside (**169**) (Zaki et al. 2017). From the aerial parts of the South American *Panicum laxum* Sw., some cyclotides such as panitide L1 (**170**) were found to be cytotoxic to HeLa cell line (Nguyen et al. 2013). From the seeds of *Panicum miliaceum* L. (purchased in India), vanillin (**171**) (a simple phenolic) was found to be weakly cytotoxic to colon cancer cell line at the concentration of 250 µg/mL with DNA fragmentation, cell cycle arrest in the G0/G1 phase, and apoptosis (Ramadoss and Sivalingam 2020). It is not known for sure whether *P. palmifolium* is toxic, but Merrill (1943) notes that the seeds are used as a famine food in the Philippines. More experiments are needed.

### **Plagiostachys albiflora** ridl. (Zingiberaceae)

This cultivable ginger is employed as a medicinal food (Table 1 and 2), and as a member of the Zingiberaceae family, it may represent a promising candidate for nutraceutical development. Its chemotherapeutic properties need to be studied, as well as its toxicity. Note that plants from the genus *Plagiostachys* Ridl. (1899) do not appear to have been the subject of any phytochemical or pharmacological studies.

### **Ampelocissus polita** (miq.) Pelser

**Figure 32.**
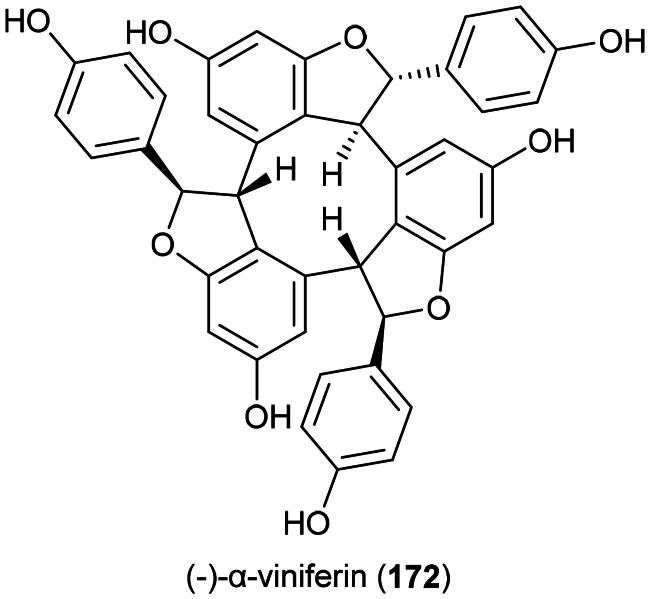
Cytotoxic stilbenoid from plants of the genus *Ampelocissus.*

This vine is used as a medicinal vegetable (Table 1). It appears to have not been the subject of any phytochemical, pharmacological, or toxicological studies. It should be noted that *Ampelocissus martini* Planch is used as a vegetable in Thailand, which could, *prima facie*, indicate that these plants are harmless. An aqueous extract of the aerial parts of *Ampelocissus latifolia* (Roxb.) Planch. (collected in India) was found to be cytotoxic to Dalton lymphoma cell line (IC_50_: 16 µg/mL), induced DNA fragmentation, and apoptosis (Chaudhuri and Ray 2020). Plant in the genus *Ampelocissus* Planch. (1884) produce cytotoxic stilbenoids such as (–)-α-viniferin (**172**) (Huang et al. 2021; Thanasansurapong et al. 2022) (Figure 32).

### **Galearia fulva** (Tul.) Miq. (Pandaceae)

This small shrub of the primary forest is used as medicinal food (Table 1). Nothing is known about the toxicity, phytochemistry, and pharmacology of this plant. A methanol extract of the leaves (collected in Malaysia) showed no toxicity to Vero cell line (Rizwana et al. 2010).

### **Melastoma beccarianum** Cogn. (Melastomataceae)

**Figure 33.**
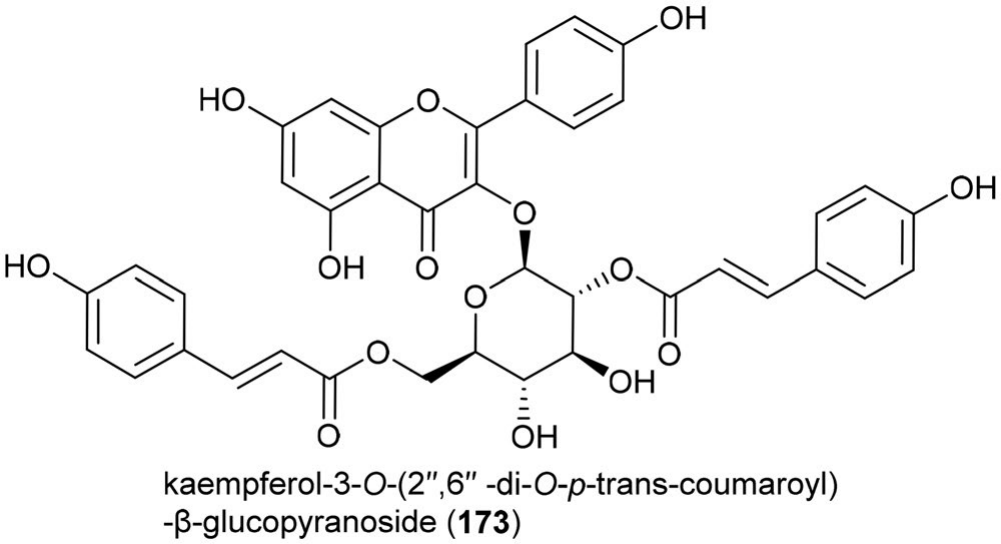
Cytotoxic flavone glycoside from plants of the genus *Melastoma.*

This shrubby endemic plant does not appear to have been the subject of toxicological, phytochemical, or cytotoxic studies. The cytotoxic principles of the genus *Melastoma* L. (1753) are phenolic in nature. Examples include naringenin (**156**) and kaempferol-3-*O*-(2ʺ,6ʺ-di-*O*-*p*-*trans*-coumaroyl)-β-glucopyranoside (**173**) from the flowers of *Melastoma malabathricum* L. (collected in Malaysia), which inhibited the growth of MCF-7 cell line with IC_50_ values of 1.3 and 0.2 µM, respectively (Susanti et al. 2007) (Figure 33).

### **Mangifera pajang** Kosterm (Anacardiaceae)

The fruits of this wild mango tree, endemic to Borneo and cultivable (Table 1 and 2), are edible (John et al. 2025) and used as medicinal food (Maid et al. 2017) (Table 1). An ethanolic seed extract inhibited the proliferation of MCF-7 and MDA-MB-231 cell lines with the IC_50_ values of 23 and 30.5 μg/mL, respectively. For MCF-7 cell line, the extract induced apoptosis *via* activation of caspases-2, −3, −6, and −8 (Bakar et al. 2010). Likewise, a seed extract (collected in Malaysia) was found to be cytotoxic to colon cancer cell line (IC_50_: 63 μg/mL) (Fadzelly Abu Bakar et al. 2010), which contained methyl gallate (**59**) that inhibited the growth of MCF-7 cell line, (IC_50_: 81 μM), induced cell cycle arrest in G0/G1 and prompted oxidative damages (Yazan et al. 2022).

Consumption of fruit juice for nine weeks improved the plasma antioxidant capacity of subjects (Ibrahim et al. 2013). These results suggest that these fruits may have potential as nutraceuticals to prevent or improve the health of cancer patients; however, further clinical trials are necessary.

### **Canarium littorale** Bl. (Burseraceae)

The fruits of this tree from the swamp forests are consumed for medicinal purposes (Table 1), but their toxicology, phytochemistry, and pharmacological properties remain unknown. Polar organic extracts of plants in the genus *Canarium* L. (1754) demonstrated cytotoxic activities as in the acetone extracts of the stem bark of *Canarium odontophyllum* Miq. (collected in Sarawak) (HCT-116; 50 µg/mL) (Basri et al. 2015; 2016; Ishak et al. 2023) and an ethanol extract of bark of *Canarium ovatum* Engl. (Balbuena et al. 2019).

**Figure 34.**
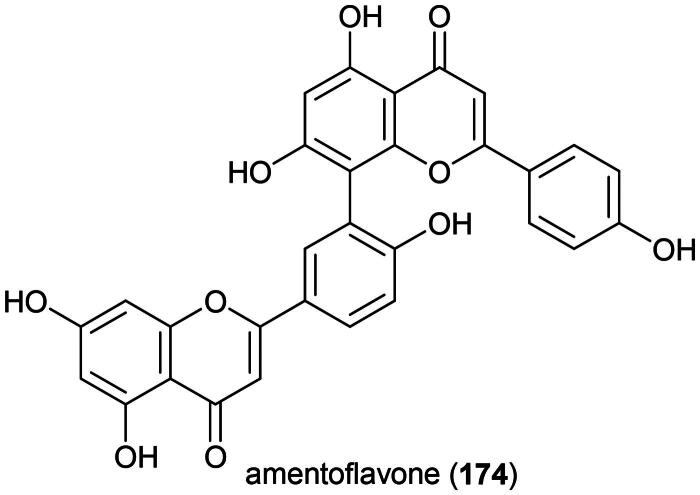
Cytotoxic biflavone from plants of the genus *Canarium.*

The biflavone amentoflavone (**174**) and protocatechuic acid (**132**) were isolated from the fruits of a plant of the genus *Canarium* L. (1759) (collected in Taiwan) (Kuo et al. 2019). Amentoflavone (**174**) was found to be cytotoxic to HCT-116 cell line (IC_50_ ≈ 100 µg/mL) (Fang et al. 2025) (Figure 34) and administered to mice xenografted with Ehrlich ascites tumor cell line, enhancing natural killer cell line (NK) activity (Guruvayoorappan and Kuttan 2007). Furthermore, administration of amentoflavone (**174**) to mice (100 mg/kg/week) xenografted with colon cancer cell line (CT26) prompted the reduction of tumor volume (Fang et al. 2025). Orally administered protocatechuic acid (**132**) prevented tumor formation in mice exposed to various types of carcinogens (Tanaka et al. 2011).

### **Dacryodes incurvata** (Engl.) H.J. Lam (Burseraceae)

The fruits of this primary forest tree are consumed for medicinal purposes (Table 1). These fruits do not appear to have been the subject of toxicological, phytochemical or pharmacological studies. An aqueous extract of *Dacryodes edulis* (G.Don) H.J.Lam leaves (collected in Cameroon) administered orally (100 mg/kg/day) for two weeks inhibited the growth of breast tumors (by 42%) induced by 7,12-dimethylbenz[*a*]anthracene in rats (Mvondo et al. 2021). An ethanol extract of fruit peels (collected in Kalimantan) was found to be cytotoxic to T-47D epithelial cell line (IC_50_: 143 ppm) (Widyanto et al. 2021).

### **Nephelium uncinatum** radlk. ex leenh (Sapindaceae)

**Figure 35.**
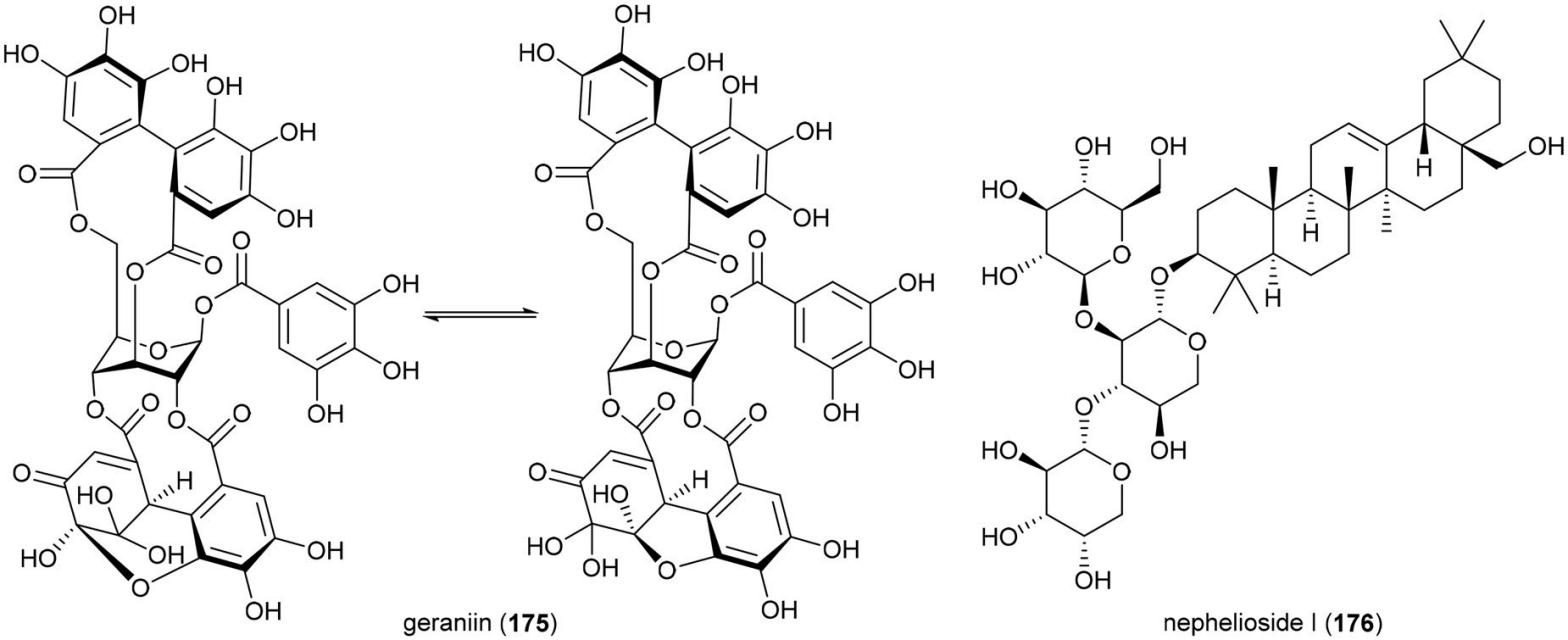
Cytotoxic ellagitannin and triterpene saponin from plants of the genus *Nephelium.*

The fruits of this cultivable tree are consumed for medicinal purposes by the Dusun (Table 1 and 2) but also by the Dayaks of East Kalimantan (Matius et al. 2018). These fruits do not appear to have been the subject of toxicological, phytochemical or pharmacological studies. An aqueous extract of *Nephelium ramboutan-ake* (Labill.) Leenh. was found to be cytotoxic to HT-29 cell line (IC_50_: 16.6 µg/mL) and induced apoptosis with DNA fragmentation, mitochondrial dysfunction, increased reactive oxygen species, increased Bax protein expression, and induced activation of caspase-3, −7 and −9 (Chan et al. 2012). Aqueous extract of peels of *Nephelium lappaceum* L was found to be cytotoxic to MCF-7 cell line (IC_50_: 94.1 µg/mL) (Jantapaso and Mittraparp-Arthorn 2022). *N. lappaceum* is also known to produce geraniin (**175**) (an ellagitannin) (Figure 35) (Abdul Ahmad et al. 2017), which at concentrations of 80 µM, inhibited the viability of glioblastoma U87MG and LN229 cell line by about 50% and induced apoptosis with increased expression of caspase-3. In mice xenografted with U87MG, geraniin (**175**) given at a dose of 60 mg/kg/day for 20 days caused a decrease in tumor weight by 44% (Ren et al. 2017). Saponins such as nephelioside I (**176**) with weak cytotoxic activities (Lu1 ED_50_: 19.5 µg/mL; LNCaP, MCF-7 and HUVEC >20 µg/mL) were isolated from the bark of *Nephelium maingayi* Hiern collected in Indonesia (Ito et al. 2004).

### **Embelia dasythyrsa** miq. (Primulaceae)

**Figure 36.**
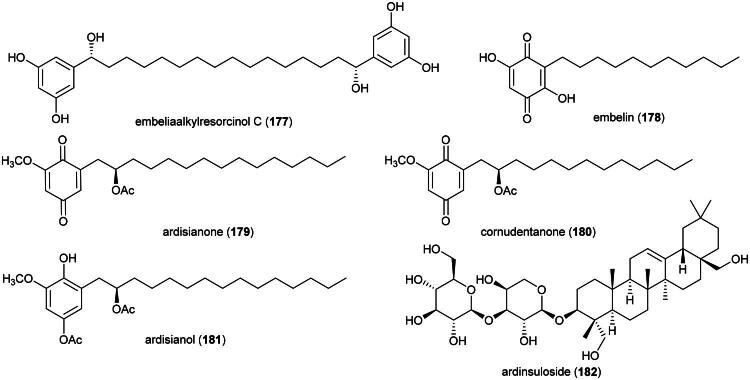
Cytotoxic compounds from plants of the genus *Embelia.*

This vine is used as a medicinal food and for fever and the Dusun even eat the leaves raw (personal observation) (Table 1). It does not appear to have been the subject of toxicological, phytochemical, or pharmacological studies. It probably contains long-chain cytotoxic alkyl benzoquinones and alkyl resorcinols, which are often produced by plants of the genus *Embelia* Burm.f. (1768) and the genus *Ardisia* Sw. (1788). Examples include embelia-alkylresorcinols C (**177**) from *Embelia ribes* Burm.f. (Chen et al. 2018), as well as embelin (**178**) (MCF-7; IC_50_: 80 µg/mL) (Kaur et al. 2015), which induced apoptosis in PC-3 cell line through downregulation of the Akt/mTOR/S6K1 pathway (Kim et al. 2013) (Figure 35). Ardisianone (**179**), cornudentanone (**180**), and ardisianol (**181**) from *Ardisia virens* Kurz were found to be cytotoxic to MCF-7 cell line (Chang et al. 2009) (Figure 36).

Other cytotoxic constituents found in plants of the genus *Ardisia* are oleanane-type triterpenoid saponins. Such compounds have been identified in *Ardisia insularis* Mez, including ardinsuloside (**182**), which inhibits the growth of A-549, HT-29, and ovarian carcinoma cell line (OVCAR) with IC_50_ values of 8.5, 16.4, and 13.6 μM, respectively (Van et al. 2015). More experiments are needed.

### **Chassalia chartacea** craib (Rubiaceae)

**Figure 37.**
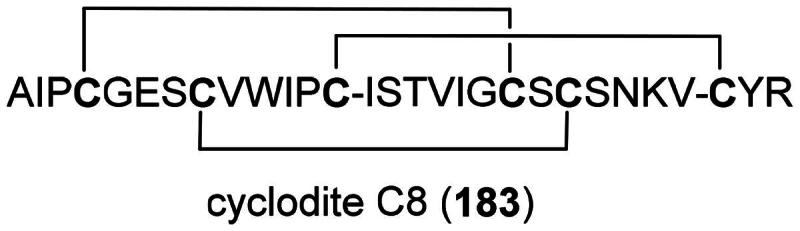
Cytotoxic cyclotide from plants of *C. chartacea.*

What is known about the phytochemistry and anticancer properties of this shrub remains very limited. The presence of cytotoxic chassatide-type cyclotides, such as cyclodite C8 (**183**) (Nguyen et al. 2012) (Figure 37), has been reported. A methanolic extract has also been found to be cytotoxic to brine-shrimp (LC_50_: 27.8 µg/mL) (Runa 2019). An alkaloid extract from the roots induced apoptosis of A549 cell line (IC_50_: 8.2 µg/mL) with cell cycle arrest in the sub-G0 phase (Gopal et al. 2022). The plant produces indole alkaloids (Schinnerl et al. 2012), whose cytotoxic effects could be evaluated.

### **Neonauclea gigantea** (valeton) merr. (Rubiaceae)

**Figure 38.**
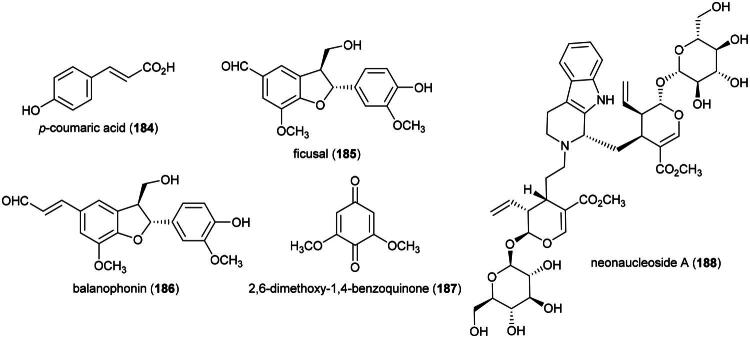
Cytotoxic natural products from plants of the genus *Neonauclea.*

This endemic tree does not appear to have been the subject of toxicological, phytochemical, or pharmacological studies. Plants of the genus *Neonauclea* Merr. (1915) are producers of cytotoxic phenolic principles. Examples are *p*-coumaric acid (**184**) (a phenylpropanoid), ficusal (**185**) (a 2-aryldihydrobenzofuran neolignan), and balanophonin (**186**) (a 2-aryldihydrobenzofuran neolignan) (Figure 38) from the stems of *Neonauclea reticulata* (Havil.) Merr. (collected in Taiwan), which were found to be cytotoxic to hepatocellular carcinoma cell line (Hep3B) with IC_50_ values of 85.3, 92.6, and 29.1 µg/mL, respectively (Chang et al. 2018). 6-Dimethoxy-1,4-benzoquinone (**187**) from the bark of *Neonauclea purpurea* (Roxb.) Merr. (collected in Vietnam) was toxic to Vero cell line (IC_50_: 1.1 µM) (Karaket et al. 2012).

Plants of this genus produce monoterpene indole alkaloid glycosides such as neonaucleoside A (**188**) from *Neonauclea sessilifolia* (Roxb.) Merr. (Itoh et al. 2003).

### **Psychotria gyrulosa** stapf (Rubiaceae)

**Figure 39.**
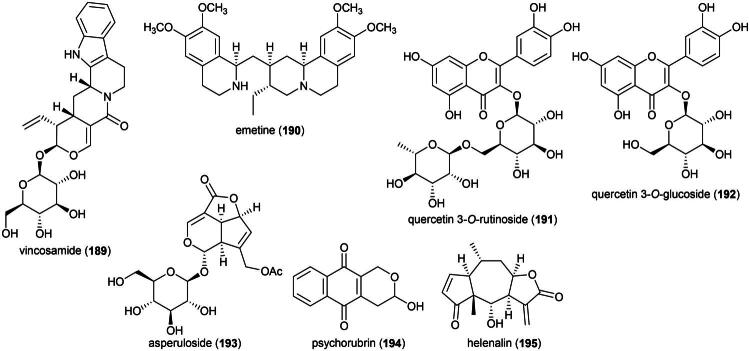
Cytotoxic natural products from plants of the genus *Psychotria.*

Plants of the genus *Psychotria* L. (1759) produce clinically useful alkaloids such as vincosamide (**189**) (Figure 39) from *Psychotria leiocarpa* Cham. & Schltdl. which inhibited hepatoma cell line (HLE) growth by about 60% at the concentration of 80 µg/mL and induced apoptosis with mitochondrial dysfunction, increased expression of Bax, decreased expression of Bcl-2, increased expression of caspase-3, and decreased expression of phosphorylated Akt. This alkaloid administered intraperitoneally at a dose of 10 mg/kg/day for 21 days to mice xenografted with hepatocellular carcinoma cell line (Bel 7402) cell line, caused a decrease in tumor volume from about 1200 to 400 mm^3^ (Zhu et al. 2022). We can also mention emetine (**190**) (a quinoline alkaloid), from *Psychotria ipecacuanha* (Brot.) Stokes, which inhibited the growth of T cell leukemia (Jurkat) (IC_50_: 0.1 µM) and induced apoptosis with DNA fragmentation, mitochondrial dysfunction, and activation of caspase-3 (Möller and Wink 2007). Emetine was able to increase the effectiveness of cisplatin on Jurkat cell line (Möller et al. 2007). It is a molecule used in therapy as an antiprotozoal agent and may have clinical application for the treatment of cancer.

Other cytotoxic principles are quercetin 3-*O*-rutinoside (**191**), quercetin-3-*O*-glucopyranoside (**192**) (flavonol glycosides), and asperuloside (**193**) (an iridoid glycoside) (HT-29; IC_50_ ˂ 50 µM) from the leaves of *Psychotria luzoniensis* (Cham. & Schltdl.) Fern.-Vill., (Ramil et al. 2021), psychorubrin (**194**) (a naphthoquinone) (KB; IC_50_: 3 µg/mL), helenalin (**195**) (a sesquiterpene lactone) (T-47D; IC_50_: 4.6 µM) (Barkhordari et al. 2023), and cyclotides from *Psychotria leptothyrsa* Miq. (collected in Hawaii) (Gerlach et al. 2010). Although plants in this genus are generally not aromatic, the essential oil of the leaves of *Psychotria asiatica* L. (collected in Vietnam) was found to be cytotoxic to lung adenocarcinoma cell line (SK-LU-1; IC_50_: 39.7 µg/mL (Tran et al. 2025). Methanol and ethyl acetate extracts of *Psychotria serpens* L. (collected in Taiwan) inhibited the growth of KB cell line (IC_50_: 20 µg/mL) (Lee et al. 1988) and HepG2 cell line (Wang et al. 2020). The toxicity, phytochemistry, and pharmacological properties of *P. gyrulosa*, a shrub endemic to the rainforest of Borneo, have not been studied.

### Other plants used by the Dusun

*Kadsura lanceolata* King has not been the subject of any phytochemical, pharmacological, or toxicological studies

## Plants used by the Rungus (Bornean group, Dusunic family)

### **Guioa bijuga** (hiern) radlk (Sapindaceae)

This coastal tree serves as a medicinal food source for the Rungus (Table 1). It does not seem to have been the subject of any toxicological, phytochemical, or pharmacological studies. Extracts from some plants in the genus Guioa Cav. (1798) have shown cytotoxic activities (Balunas et al. 2006).

### **Ixora capillaris** bremek

This endemic shrub does not appear to have been the subject of any toxicological, phytochemical, or pharmacological studies. Extracts of flowers of *Ixora javanica* (Blume) DC were found to be cytotoxic to DLA and Ehrlich ascites tumor cell line at a concentration of 12 and 65 μg/mL, respectively, while being less toxic to normal lymphocytes (Nair and Panikkar 1990) and given orally at a dose of 100 mg/kg inhibited the growth of soft tissue fibrosarcoma induced by 20-methylcholanthrene (Nair et al. 1991). Other examples of extracts with cytotoxic activities are a chloroform extract of flowers of *I. coccinea* (collected in Brazil) (HL-60; IC_50_: 36.9 μg/mL) (da Silva et al. 2016) and an extract of stems of *Ixora brevifolia* Benth was found to be cytotoxic to glioma (U251) and K562 cell line with IC_50_ values of 28.6 and 28 μg/mL, respectively (Medina et al. 2018).

**Figure 40.**
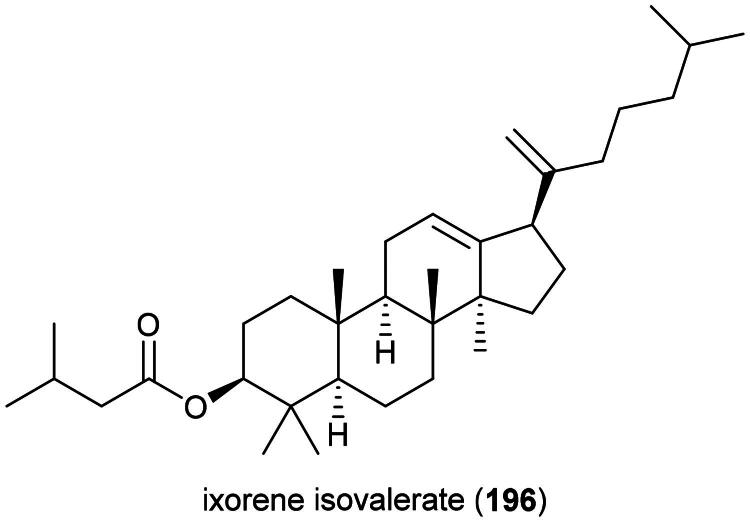
Cytotoxic triterpene from plants of the genus *Ixora.*

A hexane extract of *Ixora coccinea* L. (collected in India) inhibited the growth of Dalton’s lymphoma cell line (DLA) (IC_50_: 25 μg/mL), with inhibition of DNA synthesis. Given intraperitoneally at a dose of 200 mg/kg to mice xenografted with DLA cell line, this extract caused an increase in life span by 59.2% (Latha and Panikkar 1998). The flowers of *I. coccinea* contain ixorene isovalerate (**196**) (a dammarane-type triterpene) (Figure 40) which abrogated the survival of HeLa cell line (Ikram et al. 2016).

## Plants used by the murut (bornean group, murutic family)

### General observations

Regarding the medicinal plant species used by the Murut with possible chemotherapeutic potential, the following observations can be made: (i) about half of them are endemic, (ii) these plants are often from primary rainforest.

### **Eusideroxylon zwageri** teijsm. & binn. (Lauraceae)

**Figure 41.**
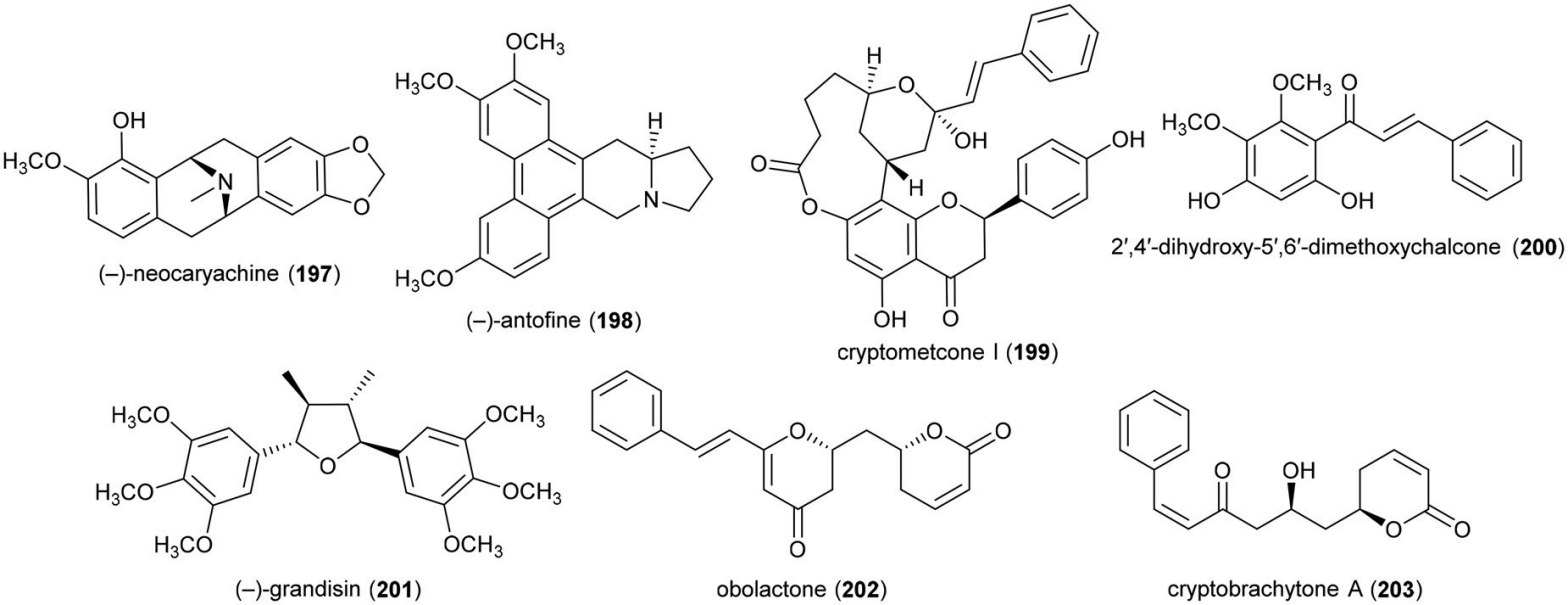
Cytotoxic natural products from plants of the genus *Cryptocarya.*

This timber tree does not seem to have been phytochemically or pharmacologically studied. It is used for the preparation of blow-gun darts, poisons (Kulip, 2003), suggesting the occurrence of bisbenzylisoquinoline alkaloids. An extract of bark was found to be cytotoxic to T-47D cell line (IC_50_: 237.5 µg/mL) (Kurniawan et al. 2023). This plant is phylogenetically close to plants in the genus *Cryptocarya* R.Br. (1810) where various classes of cytotoxic natural products have been identified. From the bark of *Cryptocarya laevigata* Blume was characterized (–)-neocaryachine (**197**) (a pavine alkaloid), toxic to multidrug-resistant cervical cancer cell line (KB-VIN) (IC_50_: 0.2 µM), and caused DNA damages and apoptosis (Suzuki et al. 2017) (Figure 41). (–)-Antofine (**198**) (a phenanthroindolozidine alkaloid) isolated from the wood of *Cryptocarya chinensis* (Hance) Hemsl. was found to inhibit the growth to ileocecal adenocarcinoma cell line (HCT-8) (IC_50_: 0.001 µg/mL) (Wu et al. 2012). Other cytotoxic principles in this genus include flavanones such as cryptometcone I (**199**) from *Cryptocarya metcalfiana* C.K. Allen (He et al. 2022), chalcones such as 2,4′-dihydroxy-5′,6′-dimethoxychalcone (**200**) (P388; IC_50_: 5.7 µM) from *Cryptocarya costata* Blume (Usman et al. 2006), (–)-grandisin (**201**) (a lignan) from *Cryptocarya crassinervia* Miq. (Saad et al. 1991), and α-pyrones such as obolactone (**202**) from *Cryptocarya obovata* R.Br. (Dumontet et al. 2004), and arylalkenyl α,β-unsaturated δ-lactones such as cryptobrachytone A (**203**) from Cryptocarya brachythyrsa H.W.Li (Fan et al. 2019). *E zwageri* is cultivable (Table 2). Phytochemical and pharmacological studies on this tree are necessary.

### **Millettia nieuwenhuisii** J.J. Smith (fabaceae)

**Figure 42.**
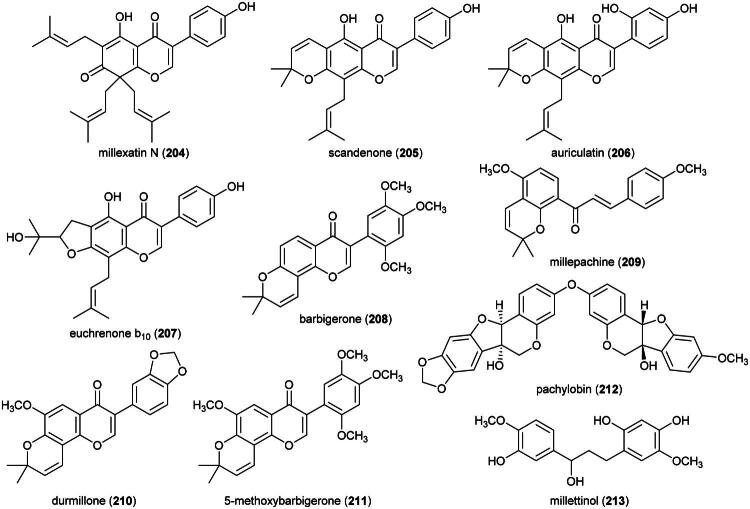
Cytotoxic phenolic compounds of plants in the genus *Milletia.*

This endemic climber of the rainforest has not been studied. It is used to treat thrush, which may indicate the presence of a cytotoxic compound, as anticandidal agents are often active against cancer cell line (Routh et al. 2011). Plants of the genus *Millettia* Wight & Arn. (1834) are a source of prenylated isoflavones with cytotoxic properties. We can cite millexatin N (**204**), scandenone (**205**), and auriculatin (**206**) from the young twigs of *Milletia extensa* Benth. ex Baker f. (collected in Thailand), which were found to be cytotoxic to MDA-MB-231 cell line with the IC_50_ values of 15.4, 13.9, and 15.3 µM, respectively (Cheenpracha et al. 2022) (Figure 42). From *Milletia pachycarpa* Benth. (collected in Thailand), euchrenone b_10_ (**207**) inhibited the growth of K562 cell line (IC_50_: 15.1 μM) (Suthiphasilp et al. 2022). From the seeds of *M. pachycarpa*, barbigerone (**208**) and millepachine (**209**) (a prenylated chalcone) were cytotoxic to HepG2 cell line with IC_50_ values of 0.6 and 1.2 µM, respectively (Ye et al. 2012). From the stems of *Millettia pachyloba* Drake, (collected from China) was isolated durmillone (**210**), toxic to HeLa and MCF-7 cell line with IC_50_ values of 6 and 11 µM, respectively. At the concentration of 20 µM, durmillone (**210**) induced apoptosis with cleavage of poly (ADP-ribose) polymerase in HeLa and MCF-7 cell line and induced cellular autophagy (Yan et al. 2019). Durmillone (**210**) isolated from *Millettia dura* Dunn, was found to be cytotoxic to A549 cell line (IC_50_: 6.6 μM) while nontoxic to lung fibroblasts (CCD19Lu) cell line (IC_50_ > 100 μM) (Buyinza et al. 2021). From the seeds of *M. pachyloba*, 6-methoxybarbigerone (**211**) and pachylobin (**212**) inhibited the growth of KB cell line with IC_50_ values of 2 and 17.6 µM, respectively (Mai et al. 2010).

Other types of cytotoxic principles in the genus *Millettia* are phenolic compounds such millettinol (**213**) (BCA-1; IC_50_: 3.4 μg/mL) from *Millettia leucantha* Kurz (Kurz) Z.Q.Song (Rayanil et al. 2011), as well as oleanane triterpene saponins (Pertuit et al. 2022). Phytochemical and pharmacological studies on *M. nieuwenhuisii* are necessary.

### **Shorea parvistipulata** F. Heim (Dipterocarpaceae)

**Figure 43.**
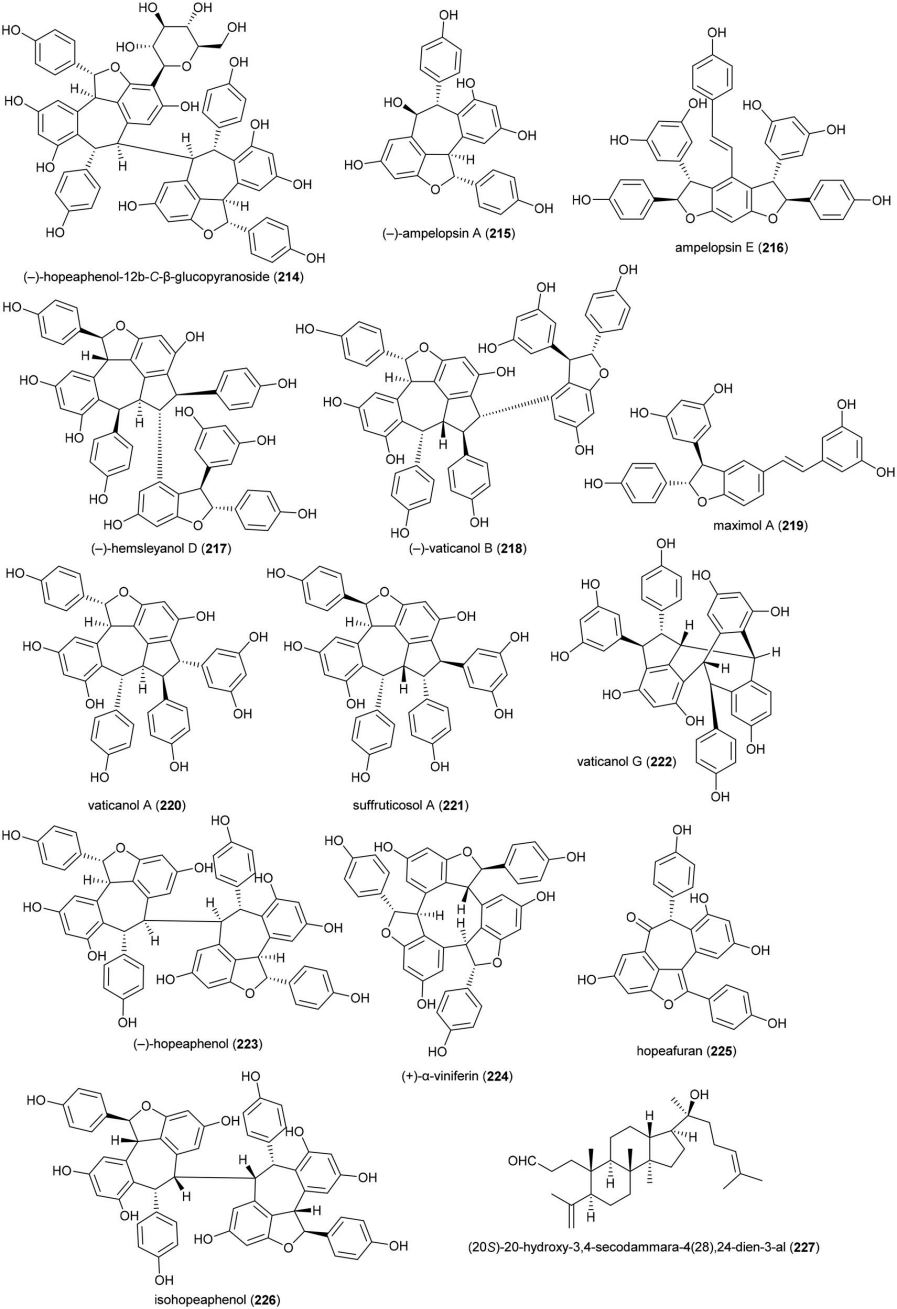
Cytotoxic stibenoids and triterpenes of plants in the genus *Shorea.*

Cytotoxic compounds have not been identified from this endemic timber tree. Plants in the genus *Shorea* Roxb. ex C.F. Gaertn. (1805) are known to produce cytotoxic stilbenoids. The bark of *Shorea gibbosa* Brandis (collected in Indonesia) contain (–)-hopeaphenol-12b-*C*-β-glucopyranoside (diptoindonesin F) (**214**), (–)-ampelopsin A (**215**), ampelopsin E (**216**), and (–)-hemsleyanol D (**217**), (–)-α-viniferin (**172**), and (–)-vaticanol B (**218**) (Figure 43), which were cytotoxic to P388 cell line with IC_50_ values of 34.6, 17, 15.3, 94.7, 25.7, and 46.4 µM, respectively (Saroyobudiono et al. 2008) (Figure 43). From the stem bark of *Shorea maxwelliana* King, maximol A (**219**), vaticanol A (**220**), suffruticosol A (**221**), and vaticanol G (**222**) inhibited the growth of HL-60 cell line with IC_50_ values ranging from 2.7 to 78 µg/mL (Zawai et al. 2013). (–)-Ampelopsin A (**215**) and (–)-hopeaphenol (**223**) from the stem bark of *Shorea hopeifolia* (F.Heim) Symington (collected in Malaysia) were active against HepG2 cell line with IC_50_ values of 22.5 and 4.5 µg/mL, respectively (Rohaiza et al. 2011). From the bark of *Shorea roxburghii* G.Don, (−)-hopeaphenol (**223**), (–)-vaticanol B (**218**), (–)-hemsleyanol D (**217**), (+)-α-viniferin (**224**) (enantiomer of **172**), and resveratrol (**123**) inhibited the growth of SK-MEL-28 cell line with IC_50_ values of 3.6, 16.6, 15.5, 7.1, and 21 μg/mL, respectively (Moriyama et al. 2016). Other examples are hopeafuran (**225**) (P388; IC_50_: 112.6 µM) (Sahidin et al. 2005), and isohopeaphenol (**226**) from *Shorea roxburghii* G.Don (Patcharamun et al. 2011; Ninomiya et al. 2017).

Other cytotoxic principles are dammarane-type triterpenes found in the resin of *Shorea javanica* Koord. & Valeton, which included (20*S*)-20-hydroxy-3,4-secodammara-4(28),24-dien-3-al (**227**) that protected mice against skin tumor formation (Ukiya et al. 2010). An ethyl acetate extract of *Shorea roxburghii* G.Don flowers (collected in Thailand) was found to be cytotoxic to AGS cell line (IC_50_: 35.5 μg/mL) (Janthamala et al. 2024).

### **Dissochaeta monticola** Bl. (Melastomataceae)

Despite *Dissochaeta monticola* being used to make blowgun dart poison, implying the potential presence of neurotoxic alkaloids, this climber, and indeed the entire genus *Dissochaeta* Blume (1831), has not been phytochemically or pharmacologically studied. This genus belongs to the tribe Dissochaeteae, which is phylogenetically close to the tribe Melastomateae (Kartonegoro et al. 2021).

### **Praravinia suberosa** (Merr.) bremek (rubiaceae)

This endemic rainforest tree has not been studied, and the same appears to apply to the whole genus *Praravinia* Korth. (1842). Nevertheless, this genus is phylogenetically close to the genus *Urophyllum* Jack ex Wall. (1824) (Koizumi and Nagamasu, 2016) where alkaloids are present (Teo et al. 1990). An ethanol extract of leaves of *Urophyllum arboreum* (Reinw. ex-Blume) Korth. was found to be cytotoxic to MCF-7 cell line (IC_50_: 136.3 µg/mL) (Jumaryatno et al. 2022).

### Other plants used by the Murut

*Goniothalamus woodii* Merr. (Annonaceae) and *Jasminum aculeatum* Blco remain unstudied.

## Ecological and ethnological considerations, and possible cultivation

The majority of these plants—with development potential either as a source of anticancer products or with the potential to be developed as nutraceutical or medicinal products—are rare species, originate from primary forests and form part of the traditional pharmacopeia of the Dusun, and to a lesser extent, that of the Murut (Table 2). The Dusun are the largest community in Sabah, followed by the Kadazan, Bajau, and Murut (Reid 1997). Native to Sabah, they are colloquially referred to as “people of the land” or “people of the orchard.” They are skilled farmers with a deep and respectful connection to the land. The Murut, or “people of the hills,” live primarily in southwest Sabah near primary rainforests and rivers (Prentice 1969), as well as in the uplands of southern Sabah (Kulip 2003). They have extensive knowledge of primary rainforests (Ahmad and Holdsworth 1994). Although cultivation information is lacking for most of these plants, some species with nutraceutical potentials can be cultivated, such as *H. zeylanica* (Un, 2010), *P. tumefacta*, *M. platytyrea*, and *M. pajang* (Tinggal and Tee, 1994) (Table 2).

## Conclusions and future direction

Despite the progress made in oncology over the last few decades, the mortality rate from cancer, and in particular from pediatric cancers, due to tumors not responding to chemotherapy, remains unacceptably high. Several anticancer drugs come from the plant kingdom and it can reasonably be anticipated that the complete study of the approximately 374,000 species identified so far (Christenhusz and Byng 2016) will lead to the discovery of molecules that that could significantly reduce this disease burden. In parallel, there is a need to develop nutraceuticals whose consumption could prevent the development of tumors by, at least in part, reducing inflammation, stimulating the immune system, and/or protecting DNA against mutagens and reactive oxygen species.

In this context, we have selected, from among the 696 species of medicinal plants of Sabah recorded to date, 64 medicinal food plant species for which there are few or no phytopharmacological studies and which belong to families known to produce cytotoxic natural products. What emerges from this study can be condensed into the following major points. (i) Most of these plants are used by Bornean ethnic groups, and primarily by the Dusun and the Murut who live in areas with high plant endemism and who for hundreds of years have learned to use plants from their immediate environment to prevent and combat diseases. Although documentation efforts have already been made to document these plants, the available data are preliminary and fragmentary. Sabah’s medicinal flora is in a very precarious situation, and endemic or primary forest species are at risk of disappearing mainly due to incessant deforestation. Furthermore, modernization and Islamization leads to the loss of oral and ancestral knowledge about these plants. (ii) Most of the plants selected deserve to be studied in depth as possible sources of original natural products for the fight against cancer, and in particular the endemic species. (iii) The oral pharmacopeia of Sabah Bornean ethnic groups includes a significant number of plants consumed as food to maintain good health. Among these, *H. zeylanica*, *P. tumefacta*, *M. platytyrea*, and *M. pajang* are interesting material.

Although some of these plants have interesting activities *in vitro* and *in vivo*, there is the need of further experiments. For the plants whose active principles have been identified or even food plants more experiments are needed to confirm their possible oncopreventive or anticancer properties. It can be also mentioned here that *in vitro* cytotoxicity is not an absolute hallmark of possible anticancer application but a simple demonstration that a natural product kills a cancer cell. It is an early indication that must be used for further *in vivo* studies and should the compound be well tolerated and effective, clinical studies become necessary. In addition, toxicological data are largely absent, making translational claims premature. Furthermore, there are no available data of the variability of the plant extracts mentioned (seasonal, geographical, preparation methods) and without phytochemical standardization, reproducibility and clinical translation remain questionable.

To date, there is no complete inventory of medicinal plants in Sabah, and the total number of these plants is estimated to be well over 696 species. However, it should be noted that deforestation to make way for palm oil plantations and the gradual loss of ancestral knowledge threaten to cause the disappearance of a large number of plants. In sum, Sabah’s medicinal food plants still represents, but for how long?, a source of potential natural products and nutraceuticals for the fight against cancer. Will we let this opportunity pass us by?

## Data Availability

Data sharing is not applicable to this article as no data were created or analyzed in this research.
